# Simultaneous two- and three-photon multiplane imaging across cortical layers in freely moving mice

**DOI:** 10.1038/s41592-026-03125-7

**Published:** 2026-06-09

**Authors:** Alexandr Klioutchnikov, Damian J. Wallace, Caleb Berdahl, Adam Sugi, Juergen Sawinski, Jason N. D. Kerr

**Affiliations:** Department of Behavior and Brain Organization, Max Planck Institute for Neurobiology of Behavior - caesar, Bonn, Germany

**Keywords:** Multiphoton microscopy, Visual system

## Abstract

Head-mounted multiphoton microscopes enable imaging of activity from neuronal populations spread throughout the cortical layers in freely moving mice but so far have been restricted to recording from one cortical layer at a time. Here, combining two- and three-photon-based excitation delivered through multiple fibers, we built a head-mounted multiplane microscope enabling near-simultaneous imaging of neuronal activity from five vertically separated planes, spread across multiple cortical layers. Both excitation pathways had remote focusing mechanisms for fine axial adjustments, enabling activity recordings from the same neuronal populations over weeks in freely behaving mice. The lightweight microscope utilized an onboard, two-channel detection system designed to enable activity recordings from neuronal populations spread across visual-cortex layers in both lit and dark conditions as well as imaging activity across posterior parietal cortex layers during complex gap-crossing behaviors. We show that during gap-crossing tasks, layer 5 and 2/3 neuronal subpopulations in the posterior parietal cortex have differential pattern sequences during free decision-making.

## Abstract (web summary)

A lightweight head-mounted multiplane microscope allows simultaneous imaging from >1,800 neurons spread across the cortical layers in freely moving mice performing complex behavioral tasks, sampled over weeks.

## Main

To understand the cortical transformation of sensory-derived inputs, theoretical frameworks^1–3^ of cortical function have assigned a clear functional role to each cortical layer, on the basis of the distinct physiological^4–6^ and anatomical^7^ properties of the layer’s neuronal inhabitants^8^. Over the past few decades, numerous tools have been developed to measure activity from large numbers of neurons across different cortical layers in freely behaving animals, aiming to uncover the link between complex behavior and the underlying neuronal activity^9–16^. Large-scale extracellular electrical recordings have shown differences in cortical modulation^17^, with unparalleled temporal resolution across vast numbers of recording sites^18^. However, their inability to unambiguously identify cell types^19^ or spatial position^20^ presents challenges in correlating these recordings with connectome reconstructions of the underlying neuronal circuitry at single-cell resolution (see ref. ^21^ for a recent overview)^22^. This issue remains unsolved. Imaging genetically encoded activity indicators using two- and three-photon excitation reveals a link between neuronal activity and genetically defined neuronal subtypes^23^, reconstructed neural circuitry^24–27^ and, when used in miniaturized head-mounted microscopes, freely moving behavior^11^. Two-photon-excitation (2PE)-based^28^ head-mounted microscopes^11,14,29^ enable planar imaging of large neuronal populations^13^ and neuronal substructures^14^, and recent developments in miniature microscopes have enabled volumetric and multiplane scanning^30,31^ using electrically tunable lenses (ETLs)^32^. Because of the inherent depth limit^33^ of 2PE-based imaging in densely labeled tissue^34^, this volumetric imaging approach is limited to the upper cortical layers, with the depth-range limited by the ETL axial imaging range, and the temporal resolution divided by the number of planes when sequentially scanning (approximately 10 Hz duty cycle over 4 planes, across 180 μm). More recently, the implementation of three-photon excitation (3PE)-based fluorescent microscopy^35–37^ into head-mounted microscopes^9,10,38^ has enabled prolonged activity recordings from the deepest cortical layers of mice freely moving between lit and dark environments^10^. The excitation light properties required for 2PE- and 3PE-based imaging of fluorescent activity-indicators, brain-tissue scattering properties, attenuation from water absorption and tissue damage from heating make 2PE well suited to upper cortical layers, whereas 3PE allows functional imaging in deeper cortical layers^34^, with an optimal cross-over depth of approximately 450 μm from the surface^39^. Here we combined 2PE and 3PE^40^ with temporal multiplexing^41^ to create five imaging planes vertically separated across superficial and deep cortical layers, allowing functional imaging from >1,800 neurons per imaging session, for weeks, in freely behaving mice. Because excitation light was delivered through a single fiber for each imaging plane, enabling near-simultaneous (8 ns between planes) imaging from the five vertically separated planes^42^, we could compare simultaneously collected activity patterns of layer 2/3 and layer 5 populations from the posterior parietal cortex in mice performing gap-crossing decision-making tasks.

## Results

### Head-mounted multiplane microscope for imaging in freely moving mice

**Fig. 1:**
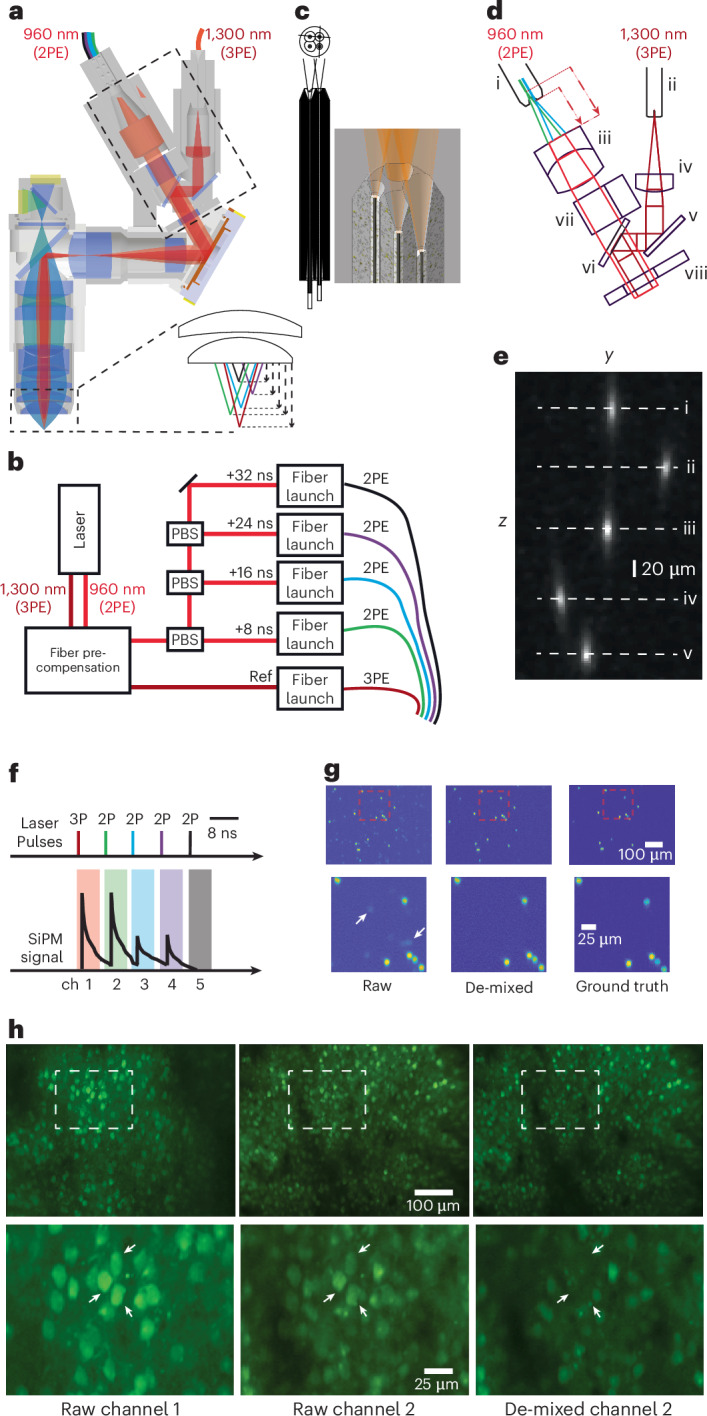
A lightweight head-mounted multiplane fiberscope for imaging across cortical layers in freely moving mice. **a**, Microscope schematic showing the 2PE and 3PE fibers, ferrule arrangement and optics used to achieve excitation of five imaging planes with axial offset (component list in Supplementary Fig. 2). **b**, Schematic layout of the optical table with the synchronized laser source for 960 nm and 1,300 nm, along with the delay structure and coupling to fibers. PBS, polarizing beam splitter. **c**, Detail of computer aided design of the 3D-printed ferrule for the four 2PE fibers. **d**, Optical layout of 2PE and 3PE microscope input paths (dashed box in **a**), showing the 2PE and 3PE ferrules (i, ii), two example 2PE beams (cyan and green), the 3PE beam (dark red), their respective collimating lenses (iii, iv), the mirrors used to combine them into the same beam path (v, vi) and the ETLs (vii, viii). The distance from each fiber tip to the collimating lens and the lens effective focal length determine the axial offset between imaging planes. **e**, Side-view of a 1 mM sulforhodamine solution showing the excitation volumes generated by beams from all fibers (2PE, i–iv; 3PE, v). The 1,300 nm 3PE beam excites sulforhodamine by 2-photon excitation. **f**, Schematic of the timing of multiplexed pulses (top), the corresponding gating for signal demultiplexing (bottom, colored boxes) and the SiPM signal (bottom, black). **g**, Average projection of a *z*-stack through a sample of 10 µm fluorescent beads imaged with laser power on all fibers (top left), the same data after de-mixing (top middle) and the same sample imaged with laser power on only one fiber (top right). Lower images show enlarged views of the regions highlighted by the red-dashed boxes. White arrows show contamination from other channels visible in the raw, but not de-mixed or ground-truth, images. **h**, Example average images of GCaMP7f-labeled neurons in primary visual cortex of a freely moving mouse from a leading channel (top left), a trailing channel (top middle) and the same trailing channel after de-mixing (top right). Bottom images show enlarged views of the regions outlined with white-dashed boxes. White arrows show neurons in the image from the leading channel (bottom left) contaminating the trailing channel raw data (bottom middle), removed after de-mixing (bottom right). All images are an average of 6,000 frames. Scale bars under top and bottom images apply to all images in the respective row.

To create a head-mounted microscope with multiple, vertically separated imaging planes for freely behaving mice, we developed a system for delivery of excitation light through multiple optical fibers, with the fiber tips positioned at different distances from the collimation lens^42^. This enabled spatially separated focal points for each fiber after the objective (Fig. 1a and Supplementary Fig. 1). We used a laser system providing synchronized pulses at two wavelengths for imaging with both 2PE (960 nm) and 3PE (1,300 nm) of GCaMP7f activity indicators^23,43^, allowing temporal multiplexing of excitation sources^41^ and parallel imaging of multiple planes. The 2PE beam was sequentially split into four channels using half-wave plates and polarization-dependent beam splitters, enabling an adjustable splitting ratio. This setup allowed for manual adjustment of pulse energy for each excitation channel. The arrival time of the 3PE pulse was used as a reference, with the arrival times of pulses for each 2P excitation channel staggered by introducing an 8-ns delay between successive channels through adjustments to the optical path lengths (Fig. 1b). Dispersion compensation for the 2PE^11,14^ and 3PE^9^ beams was implemented as the initial step in each excitation pathway, prior to 2PE beam splitting. Each beam was then transmitted to the microscope using individual fiber launchers and hollow core fibers, consisting of one 3PE fiber and four 2PE fibers (Fig. 1b). To maximize excitation efficiency, each fiber was aligned with the polarization orientation of each laser beam. Precise positioning of the 2PE fibers relative to the microscope’s optical path was achieved using high-resolution 3D-printed custom ferrules featuring a defined three dimensional (3D) position for each of the four bare fiber-tips (Fig. 1c). The fiber-tip positions were optimized to minimize lateral displacement of their focal points (and, by extension, imaging planes) while introducing sufficient separation between the fiber tips to provide 80 µm of axial spacing between two adjacent planes. Ultimately the lateral distance between the fiber tips was limited by the fiber cladding diameter. Axial spacing of 80 µm between the 2PE imaging planes was chosen to reduce optical cross-talk between adjacent planes due to the extended axial point spread function (PSF), and provided a total axial span of 240 µm sampled by the four 2PE focal volumes. The 3PE fiber was held in a separate ferrule to allow independent adjustment of the 3PE focal point relative to the four 2PE foci, with the 3PE and 2PE excitation paths being combined using two dichroic mirrors (Fig. 1d and Supplementary Figs. 1b,c and 2). To confirm the axial separation of the focal points from the five excitatory beams, we generated PSFs for all channels in a 1 mM solution of sulforhodamine 101 while imaging the resulting fluorescence with an orthogonally-mounted camera (Fig. 1e and Supplementary Video 1). Scanning all five beams using the same miniature electro-mechanical system (MEMS) scanner provided five spatially separated imaging planes. The emitted fluorescence from all planes was collected into a two-channel (green and blue) detection system based on silicon photomultipliers (SiPM). The time multiplexing introduced into the excitation laser pulses was then used to define temporally separated integration windows for each channel (Fig. 1f), with the high bandwidth of the SiPMs allowing the demultiplexing of the signal into five separate planes per color channel (Fig. 1f). To introduce remote focusing functionality, two ETLs were incorporated into the microscope design (Supplementary Fig. 2), one adjusting the position of all imaging planes and the other allowing independent positioning of only the 2PE planes relative to the 3PE plane. Because ETLs in miniature microscopes^13^ are typically not used in a configuration in which they are conjugated to the back focal plane of the objective lens, the excitation numerical aperture (NA) and the optical resolution of the microscope are not constant over the full tuning range. Here, they were incorporated for the purpose of fine adjustment of the imaging depth, mainly for the retrieval of the same neuronal populations over successive imaging sessions, with functional imaging and characterization of the microscope’s resolution all performed with the ETLs set close to the center of the range.

The weight of the resulting microscope was 2.5 g, and the maximum field of view of the microscope was 450 × 600 µm^2^, scanned at 9.73 Hz with a pixel resolution of 410 × 340 pixels. The lateral and axial optical resolution of the microscope in the configuration described above was measured as follows: 1.30 ± 0.03 µm and 14.8 ± 1.1 µm, respectively, for the 3PE plane and 1.45 ± 0.11 µm and 22.2 ± 1.8 µm, respectively, for the 2PE (mean ± s.d., all measured with a sample of 1-µm-diameter fluorescent beads) (Supplementary Fig. 3). The maximum power under the objective lens was 36 mW for the 960 nm laser and 100 mW for the 1,300 nm laser. The maximum laser power used during imaging was 36 mW for the 960 nm laser and 50 mW for the 1,300 nm laser. A second version of the microscope was also assembled, featuring a fast imaging rate with a 40 Hz frame rate, a resolution of 200 × 180 pixels and a field of view of 380 × 320 µm^2^ (Supplementary Fig. 4 and Supplementary Table 1).

### Electronic cross-talk removal

The ideal scenario for demultiplexing fluorescence signals involves fast-rising, short-lived excitation pulse-evoked photon transients, which have fully decayed before the integration window for the next channel begins. In the current miniature multiplane microscope, the detected fluorescence signal was transmitted to a remote high-speed trans-impedance amplifier over a 2-m-long extra-thin coaxial cable. The SiPM detector’s behavior, coupled with long transmission lines, produced slower photon transients than can be achieved with equivalent size-unlimited photo multiplier tubes^40^ with high-bandwidth cables, resulting in a pronounced contamination in trailing channels from leading channels (Fig. 1g,h). Multiplexing cross-talk between channels was characterized in the current microscope by imaging fluorescent beads with a diameter of 10 µm (Fig. 1g and Supplementary Fig. 5a–c). The multiplexing cross-talk was unidirectional, originating from only the earlier channels (Supplementary Fig. 5). Because the 3PE beam was the first in the multiplexing sequence, the cross-talk affected only the 2PE planes. To de-mix the imaging channels, we developed an iterative optimization routine that minimized cross-talk between two channels by subtracting a fraction of the intensity of the leading channel from the trailing channel. To measure the accuracy of this de-mixing approach, we generated ground-truth data by first imaging a volume of fluorescent beads with all channels operating, followed by sequential imaging of the same volume with the same microscope but with only one channel operational at a time (Fig. 1g and Supplementary Fig. 5a). Average fluorescence in regions of interest (ROIs), defined on objects in the leading channels but computed in trailing channels, decreased by a factor of 4.4 ± 1.8 (mean ± s.d.) after cross-talk removal. This reduction was not significantly different when using the same metric on ground-truth data with the same ROIs (Wilcoxon’s rank-sum test; raw versus ground truth *P* value, 0.0286; de-mixed versus ground-truth *P* value, 0.2). Applied to in vivo images of neuronal populations expressing GCaMP7f (Fig. 1h), the same de-mixing approach effectively removed the cross-talk in each channel (Fig. 1h). All subsequent descriptions and quantifications of imaging data were performed on data after de-mixing to remove the multiplexing cross-talk.

**Fig. 2:**
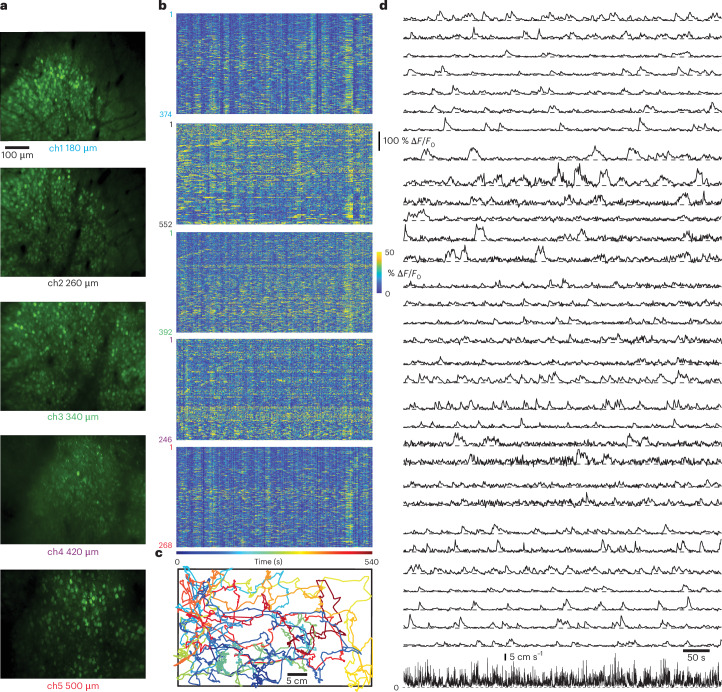
Multiplane imaging of neuronal populations in superficial and deep cortical layers in a freely moving mouse. **a**, Average images for each imaging plane of neuronal populations in the visual cortex of a freely moving mouse over an approximately 10-min imaging session with corresponding imaging depths (all images from one mouse, an example from *n* = 4 sessions from 3 mice). ch, channel. **b**, Raster representations of fluorescence dynamics corresponding to the imaging planes in **a**. Rows show data from identified ROIs. The color scale at the right of the third panel applies to all rasters. Fluorescence (*F*) data were smoothed with a moving ten-frame average. Colored numbers on the right of the rasters represent neuronal count. **c**, Animal trajectory in the arena, corresponding to the fluorescence data shown in **b**, color-coded by time. **d**, Example fluorescence traces from the neuronal populations shown in **a**,**b**. The bottom trace shows animal velocity.

To establish the utility of the head-mounted multiplane microscope for imaging neuronal activity simultaneously from multiple planes, we next imaged neuronal populations in layers 2/3 and 5 expressing GCaMP7f in freely moving mice (Fig. 2a–c, N = 4 sessions from 3 mice spread over 1 to 4 different days). The combination of microscope, head-mounted implant and mounting system design resulted in stable imaging during behaviorally active periods (median ± s.d., Euclidean image displacement before motion correction and over all planes, 2.40 ± 2.93 µm; *n* = 570,000 frames total from four sessions from three mice, Supplementary Video 2), with 80.18% of the data having frame-wise image displacement of less than 5 µm. Image motion was comparable over all imaging planes, regardless of imaging depth or excitation modality (median ± s.d., Euclidean image displacement prior to motion correction for each plane, from superficial to deep, 1.80 ± 2.51 µm (2PE), 2.03 ± 2.62 µm (2PE), 3.13 ± 3.79 µm (2PE), 1.84 ± 1.99 µm (2PE) and 3.085 ± 2.89 µm (3PE); data from *n* = 114,000 frames from 4 sessions from 3 mice in all cases). During these recordings, Ca^2+^ transients were present in labeled somas and dendrites located in all imaging planes (Fig. 2b,c).

### Stable imaging from the same neuronal populations across weeks

**Fig. 3:**
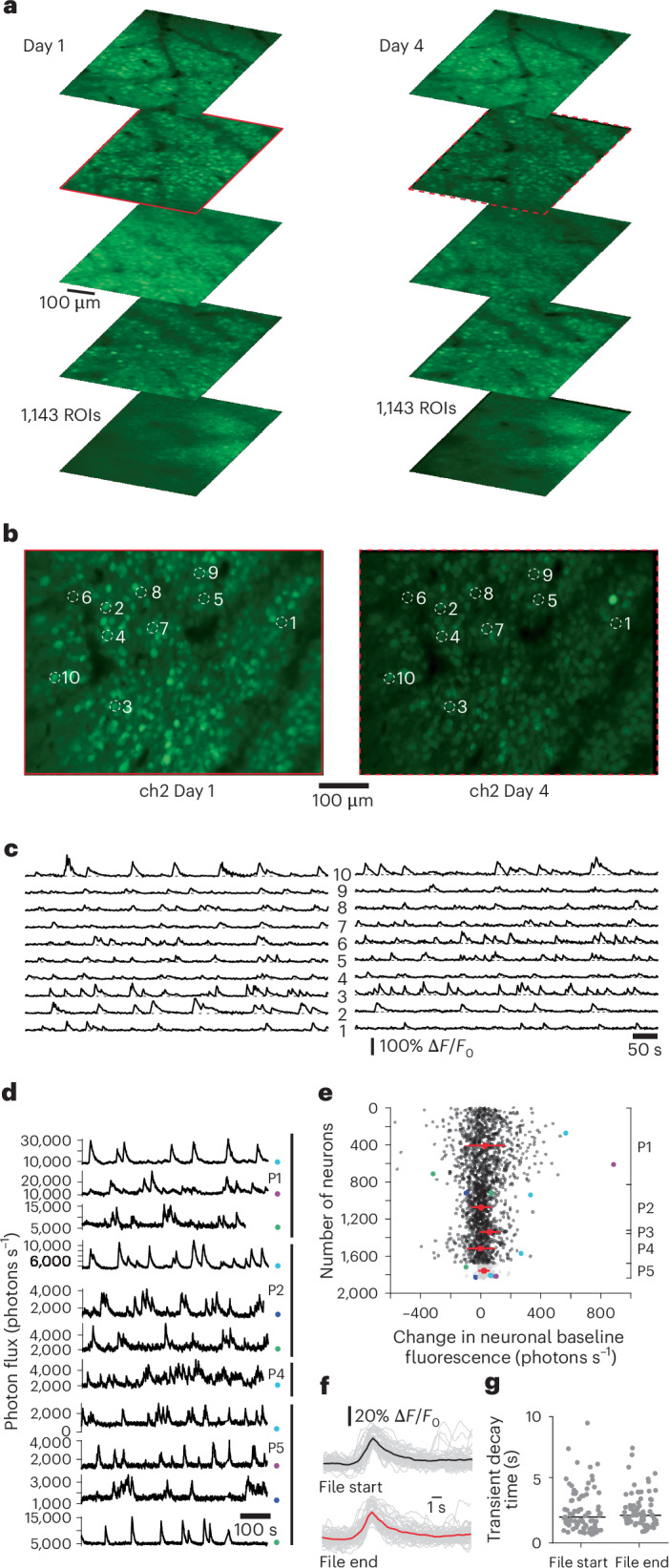
Multiplane imaging is stable over imaging sessions and multiple days. **a**, Overview of average images from all planes of labeled neurons in the posterior parietal cortex on one day of imaging (left), and the same populations imaged four days later (right). The same 1,143 ROIs have been identified in both datasets. **b**, Overview images from channel two in **a** (example dataset from *n* = 2 experiments, showing 10 example ROIs, indicated by dashed circles, from which fluorescence traces are shown in **c**). **c**, Fluorescence traces corresponding to the ROIs labeled in **b**. **d**, Example raw data traces from three 2PE planes (top, P1, P2 and P4) and one 3PE plane (bottom, P5). Color marks denote corresponding points in **e** for each plane. **e**, Scatter plot of the difference in baseline (inactive) raw fluorescence in the first and last 20% of each imaging session for all neurons in all planes (2PE, black; 3PE, gray). Data from individual planes (P1–P5) are indicated on the right. Colored points denote points corresponding to the example traces shown in **d**. Red circles and bars show the median and s.d. for each plane. Data from 4,899 neurons, from 4 datasets from 4 mice. **f**, Example transients from the first (top) and last (bottom) 20% of recordings used for the analysis of transient decay time constant in **e** (*n* = 50 transients in both cases, mean trace for first and last examples shown in black and red, respectively). **g**, Decay time constants (*τ*) calculated from exponential fits to individual transients. Data from 81 and 70 individual transients for the first and last 20% of recordings, respectively. Black bars show the median.

The total number of neurons sampled by all image planes depended strongly on the density of labeling across the cortical layers (Supplementary Fig. 6a,b). In the experiments described here, the number of neurons imaged in each of the five planes during individual sessions ranged from 123 to 552 per plane, with the average number of neurons in all imaging planes being 1,225 ± 439 neurons per session (mean ± s.d., range 782 to 1,832, *n* = 4 datasets from 3 mice). Although we manually defined ROIs for the analyses here to provide a conservative estimate of neuron counts, automated detection of ROIs was also possible (Supplementary Fig. 7). The variable splitting ratio achieved through half-wave plates and polarization-dependent beam splitters allowed for adjustment of excitation illumination such that each of the 2PE planes had similar excitation intensity, and both the signal-to-noise ratio and distribution of Ca^2+^-transient amplitudes were similar across all 2PE planes regardless of imaging depth (Supplementary Fig. 6c–e). To assess both the stability and the effects of the multiplane microscope on the tissue being imaged, we first imaged similar neurons (*n* = 1,143) from all imaging planes on two days (Fig. 3a,b). Overview images from all imaging planes enabled clear identification of matching neurons across the two datasets, collected three days apart (Fig. 3b). Similar patterns of Ca^2+^ transients were observed in the same neurons across both datasets (Fig. 3c). In a second dataset, neuronal populations exhibiting strong similarities were imaged on two occasions, 18 days apart, showing vigorous activity on both days (Supplementary Fig. 8). Together, these results suggest that the approximately 10-minute-long sessions of continuous imaging had minimal, if any, detrimental effects on the individual neurons. Both the resting (inactive) neuronal fluorescence and the decay time constant of neuronal Ca^2+^ transients increase following photodamage from excessive exposure to multiphoton excitation sources^44,45^. To quantify the influence of the multiplane microscope on the tissue during free behavior, we measured these parameters and compared their values at the start and end of the imaging sessions. There was no significant difference for any of the imaging planes between resting neuronal fluorescence at the start versus the end of a recording (Fig. 3d,e; median ± s.d., photon flux, calculated over the first and last 20% of the recording, respectively: plane 1, 337.0 ± 2003.3 photons s^−1^, 370.8 ± 1984.8 photons s^−1^, *P* = 0.69, *n* = 827 neurons; plane 2, 649.6 ± 612.8 photons s^−1^, 638.8 ± 618.6 photons s^−1^, *P* = 0.92, *n* = 493 neurons; plane 3, 971.4 ± 692.8 photons s^−1^, 1010.0 ± 708.6 photons s^−1^, *P* = 0.62, *n* = 37 neurons; plane 4, 623.2 ± 923.6 photons s^−1^, 630.2 ± 951.0 photons s^−1^, *P* = 0.90, *n* = 319 neurons; plane 5, 366.1 ± 465.5 photons s^−1^, 394.6 ± 467.3 photons s^−1^, *P* = 0.30, *n* = 162 neurons; plane 1 most superficial, *n* = 4 datasets from 3 mice, Wilcoxon’s rank-sum test in all cases), nor was there a significant difference in transient decay time constant (median ± s.d., transient decay (*τ*), start, 2.052 ± 1.718 s; end, 2.188 ± 1.406 s; *n* = 81 and 70 transients respectively, *P* = 0.36, Wilcoxon’s rank-sum test, data from 4 datasets from 3 mice). This indicates that the multiplane microscope does not induce any obvious photodamage, even during sustained and repeated imaging sessions.

To reduce the impact of the microscope and associated cabling on the animal’s behavior, we used a counterweight system, which attached to the cable bundle approximately 60 cm from the microscope along the length of the cable bundle (Supplementary Fig. 9). While carrying the microscope, the mice actively explored the behavioral arena and readily crossed a gap between two elevated platforms. During imaging recordings in the open arena, the mice spent, on average, 50.67 ± 22.49% (range, 34.78–66.57%) of the time running (velocity > 5 cm s^−1^, *n* = 2 recordings from 2 mice). While running, their average velocity was 10.0 ± 6.0 cm s^−1^ (average recording time, 577.6 ± 53.6 s, *n* = 2 recordings from 2 mice), which is similar to previous measurements taken using a head-mounted microscope^10^ (9.0 ± 0.85 cm s^−1^) and to measurements from mice carrying only LEDs for position tracking^10^ (10.72 ± 1.75 cm s^−1^). While running, their average total path length was 2,272.5 ± 1,590.1 cm (mean ± s.d.; range, 1,148.2–3,396.9 cm; *n* = 2 recordings from 2 mice). Because the microscope described here has four more optical fibers contained in the microscope’s cable bundle than do conventional single-fiber microscopes, we also tested the influence of the extended cable bundle on the animals’ behavior. For all behavioral parameters, there was no significant difference between the mice carrying the multiplane microscope and those carrying only the LEDs used for position tracking (Supplementary Fig. 9; mean ± s.d. for multiplane microscope and tracking LEDs, mean velocity 7.36 ± 1.15 cm s^−1^ and 5.91 ± 0.93 cm s^−1^, respectively, *P* = 0.23; mean running velocity 11.47 ± 0.93 cm s^−1^ and 11.44 ± 0.06 cm s^−1^, respectively, *P* = 0.86; fraction of time running 0.54 ± 0.07 and 0.43 ± 0.07, respectively, *P* = 0.23; total path length 5,176.2 ± 833.2 cm and 3,506.1 ± 677.4 cm, respectively, *P* = 0.06; *n* = 3 datasets from 3 mice for multiplane microscope, and *n* = 4 datasets from 4 mice for tracking LEDs only, Wilcoxon’s rank-sum test in all cases). There was no significant difference in behavior between animals carrying a proxy microscope with a cable bundle with five optical fibers and those carrying the same proxy microscope with only a single optical fiber (Supplementary Fig. 9; mean ± s.d. for proxy microscope with five optical fibers or one optical fiber in all cases, mean velocity 7.00 ± 0.49 cm s^−1^ and 5.97 ± 1.17 cm s^−1^, respectively, *P* = 0.67; mean running velocity 11.42 ± 0.02 cm s^−1^ and 11.23 ± 0.10 cm s^−1^, respectively, *P*= 0.33; fraction of time running 0.52 ± 0.06 and 0.43 ± 0.12, respectively, *P* = 0.67; total path length 3,990.9 ± 409.3 cm and 3,205.4 ± 964.5 cm, respectively, *P* = 0.67; *n* = 2 datasets from 2 mice and Wilcoxon’s rank-sum test in all cases). To further assess the extent to which the system impeded the animals’ head dynamics, we quantified the average absolute angular accelerations (azimuth and elevation, Supplementary Fig. 10). We compared the distributions of absolute accelerations over the 10-min sessions, as well as changes binned in 60-s intervals (Supplementary Fig. 10b,d). For absolute azimuth acceleration, there was no significant difference between mice carrying the real microscope and either the single-fiber or multi-fiber dummy (Supplementary Fig. 10 and Supplementary Table 2 for associated *P* values), although accelerations measured with the tracking tripod were only significantly faster than those measured with any of the other devices (Supplementary Fig. 10, mean ± s.d. absolute acceleration for tripod only, single-fiber dummy, five-fiber dummy and real multiplane microscope 8.44 ± 8.95, 6.54 ± 6.59, 5.99 ± 4.40 and 5.85 ± 3.69 rad s^−2^, respectively; Kruskal–Wallis test in all cases, Supplementary Table 2). For elevation, the acceleration measured for the real microscope was significantly higher than that for the other three devices, although there was no observable relationship between acceleration and complexity or weight of the device (Supplementary Fig. 10 and Supplementary Table 3 for associated *P* values). Quantified over the time course of the 10-min sessions, azimuth acceleration for the real microscope was consistently about 6 rad s^−2^ throughout; for the other conditions, there was a progressive small reduction with time (Supplementary Fig. 10b). For elevation, all devices were similar, although acceleration values measured with the real microscope were higher (Supplementary Fig. 10d). In summary, there was a small but measurable effect on rotational acceleration for mice carrying any microscope, including the multiplane microscope, but mean velocity, mean running velocity, fraction of time spent running and total path length were not significantly different.

### Linking free decision-making behavior to neuronal activity patterns across cortical layers

**Fig. 4:**
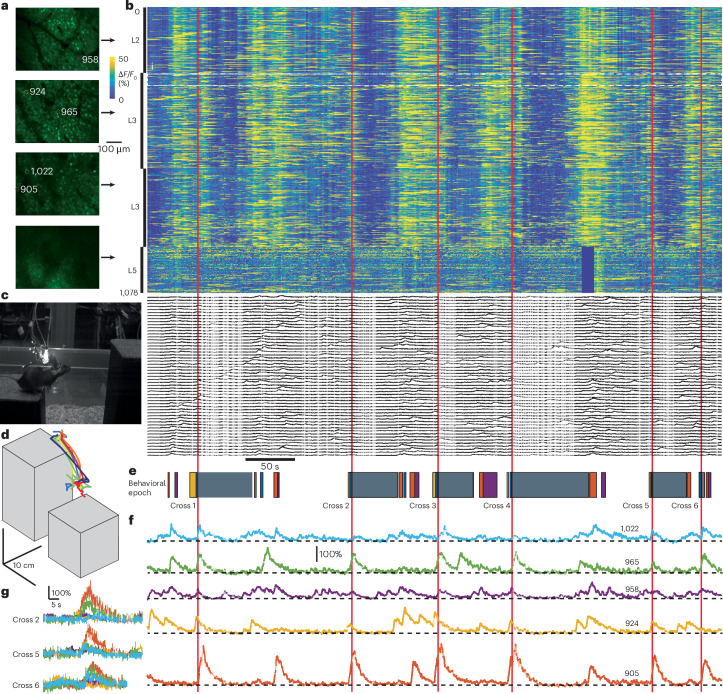
Simultaneous imaging of neuronal activity across layers in the posterior parietal cortex during repetitive complex gap-crossing behavior. **a**, Overview images of neuronal populations in the posterior parietal cortex from four planes (top in layer 2, second and third from the top in layer 3 and the bottom in L5) during the gap-crossing session in which the data in **b**–**g** were recorded. **b**, Raster representation of 1,078 ROIs identified across all four imaging planes shown in **a**. Red lines indicate crossing times. Black arrows indicate correspondence between overview average images in **a** and the relevant section of the raster representation. The dashed white box shows the 53 neurons for which fluorescence traces are shown below the raster. **c**, Image of the mouse just before jumping. **d**, Measured 3D trajectories for all identified gap crosses in the imaging session shown in **a**,**b**,**e**,**f**. Colors for crosses 1–6 are, respectively, dark blue, light blue, green, yellow, orange and red. **e**, Temporal representation of identified behavioral events in the recording session (blue, cross to top platform; orange, cross to middle platform; magenta, cross from middle platform to arena floor; gray, reward location on top platform). **f**, Fluorescence traces of five example neurons (indicated by colored dashed circles in **a**) with transients around gap crosses. **g**, Overlay of fluorescence traces from gap crosses 2, 5 and 6 for the same neurons as in **f**.

We next measured neuronal activity simultaneously from cortical layer 2/3 (L2/3) and 5 (L5) in the posterior parietal cortex of mice during complex behaviors (Fig. 4a,b and Supplementary Fig. 11). Mice were trained to estimate the distance of a gap between two raised platforms and subsequently cross from one platform to the other to receive a food reward (Fig. 4c). Neuronal populations were recorded in approximately 10-min-long files, enabling calcium transients to be continuously recorded from large populations spread across the cortical layers during multiple gap-cross attempts (Fig. 4d, mean ± s.d. file duration, 608.2 ± 9.6 s, sessions of 1–2 imaging files, *n* = 4 datasets from 2 mice). Through a combination of behavior tracking and behavioral monitoring with visible light cameras, we determined the animal’s position in the arena (Fig. 4d) and classified the animal’s behavior (Fig. 4e) throughout the task. From these classifications, we established a common behavioral point of reference for each trial to correlate neuronal activity to each in the behavior epochs (Fig. 4e,f). Because L2/3 neurons in the posterior parietal cortex display repetitive neuronal activity patterns correlated with the various epochs of decision-making behavior in mice engaged in virtual-reality-based tasks^46^, as well as in freely navigating rats^47^, we next analyzed the neuronal kinetic traces from all planes synchronized to the time that the animal landed on the target landing platform (Fig. 4f). This analysis showed that a subpopulation of L2/3 neurons was consistently active before, during and after the gap-crossing landing time for each gap-crossing trial (Fig. 4g). Because previous imaging studies have been limited to L2/3 of head-fixed mice without access to L5 neurons, we next compared the activity patterns of L2/3 with those simultaneously recorded in L5.

### Differential timing of neuronal activity patterns between the cortical layers during decision making

**Fig. 5:**
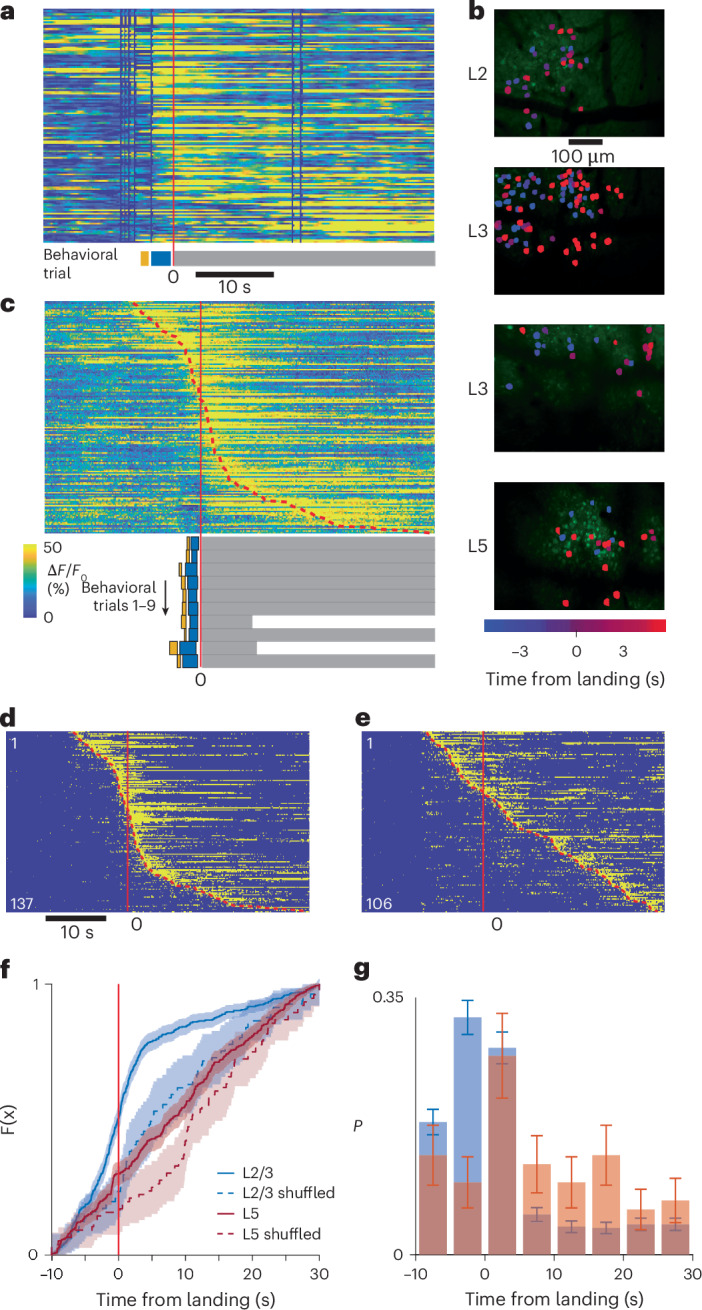
Differential neuronal activity sequence patterns between posterior parietal cortical L2/3 and L5 during decision-making. **a**, Raster representation of neuronal fluorescence traces from a single gap cross, recorded during one session from one animal, spanning 20 s before to 30 s after the cross. Traces are shown only for neurons exhibiting substantial activity onset during the specified time window, based on fluorescence traces averaged over all gap crosses, synchronized to the moment of contact with the top (reward) platform (red line). Neurons are ordered according to the timing of activity onset, from earliest at the top to the latest at the bottom. The color coding is the same as that in Figure 4e. The color scale is under the raster in **c**. **b**, Overview images for four imaging planes from the same session in which the gap cross shown in **a** occurred; an example from *n* = 2 experiments is shown. Colored ROIs represent neurons with activity onset in the time around the cross, color-coded by onset time. The color scale is shown under the bottom image. Putative cortical layers based on imaging depth are shown to the left of the images. **c**, A representation similar to that in **a**, but showing fluorescence traces averaged over all gap crosses in the session (*n* = 9 trials). The red dashed line represents the detected onsets of each neuron. Behavioral classification is the same as in **a**. **d**, A binarized display of the data in **c**. Solid and dashed red lines retain the same meanings as in **c**. **e**, The same representation as in **d**, with the timing of gap crosses randomly shuffled. **f**, Cumulative probability functions of onsets of imaged neurons in L2/3 (red solid, *n* = 602 neurons) and L5 (blue solid, *n* = 81 neurons), with corresponding functions from shuffled data for L2/3 (dashed red, *n* = 517 neurons) and L5 (dashed blue, *n* = 76 neurons). The 95% confidence interval is shaded in the corresponding color for both traces, with the line being the center. Data from all datasets from three imaging sessions from two animals. **g**, A histogram of time distribution of sequence neurons, with the same data and color code as in **f**. The error bars show the s.d., calculated under the assumption of a Poisson distribution, with the top of each histogram bar being the center.

We ranked all neurons on the basis of the timing of their activity onset relative to the behavioral reference point, in this case, the time of contact with the target platform, for individual gap-crossing trials (Fig. 5a). This analysis allowed us to establish which neuronal subpopulations in each layer were active at consistent time points of the behavioral sequence (Fig. 5b). We found that compared with L2/3 neurons, a smaller fraction of L5 neurons was active before and during the crossing events (Fig. 5b). To quantify this, we binarized the neuronal activity, fitted the activity onset for each neuron in the ranked activity sequence (Fig. 5d) and compared these results with the same data in which the neuronal activity had been temporally shuffled (Fig. 5e). Neuronal activity during these behavioral periods for both L2/3 and L5 populations differed from that in shuffled data around the landing event (*n* = 2 mice, 3 imaging sessions). The density of sequence cells in L2/3 was, on average, 2.07 and 2.22 times larger than in shuffled data for the 0–5 s before the gap crosses and the 0–5 s after gap crosses, respectively (Wilcoxon’s rank-sum test, *P* = 0.0092 and 1.56 ×10^−8^, Supplementary Fig. 12 and Supplementary Table 4), and in L5, on average, 2.95 times larger than in shuffled data for the 0–5 s after gap crosses (Wilcoxon’s rank-sum test, *P* = 0.0182, Supplementary Fig. 12 and Supplementary Table 4). In addition, comparison of the L2/3 and the L5 neuronal responses during these behavioral epochs showed a significant increase in the number of neurons active in sequences before the crossing event in L2/3, but not in L5 (Fig. 5f,g, Supplementary Fig. 12 and Supplementary Table 4 for associated *P* values). Together, these results suggest that neuronal activity in the posterior parietal cortex differs across layers during free behavior, and although L2/3 neuronal activity shows a strong repetitive temporal sequence during the entire behavioral sequence, L5 neurons are more active in the post-decision period during reward acquisition.

## Discussion

We designed and implemented a multiplane microscope enabling the measurement of neuronal activity from large cortical populations spread across the cortical layers in mice performing complex free-moving behavioral tasks. The microscope was designed to measure the same neuronal populations over days while capturing activity from neuronal populations in deep and superficial cortical layers. The total frame rate was not affected by the number of planes, but instead frames from each plane were acquired in parallel, increasing the overall data-acquisition rate by a factor equal to the number of fibers. This temporal, multiplexed, multi-fiber approach offers advantages over previous multiplane approaches using ETLs^13,30,31^, including enabling the combination of both 2PE and 3PE modalities, and their imaging advantages^34,39^, as well as enabling near-simultaneous sampling across vertically separated planes. In addition, the separate ETLs in the 2PE and 3PE pathways enabled independent adjustment of the imaging planes for each modality, allowing for localization of neuronal populations from previous imaging sessions. Finally, by combining multiple fibers with axial offsets, ETLs and 2PE and 3PE techniques, we extended the axial range between imaging planes to cover the distance between L2 and L5 of the mouse cortex, enabling simultaneous imaging of the deep and shallow cortical layers.

The high-frequency electronics required for demultiplexing the detected fluorescence, combined with the characteristics of the connections between the microscope-mounted detectors and signal digitization and amplification, made it vulnerable to ‘ghosting’ artifacts from one imaging channel to the next^40^. This cross-talk was unidirectional owing to the asymmetric time structure of the multiplexing. The laser repetition rate used here was 2 MHz, and only the first approximately 50 ns were used for imaging, providing an approximately 450-ns delay before the next pulse, which was enough for the detection electronics to settle down. If a higher repetition rate or multiplexing rate is used, the problem might become more complicated.

Although we manually defined ROIs for the datasets here to verify our de-mixing approach, the imaging data obtained after the preprocessing steps are suitable for automated detection of neuronal structures.

The addition of extra excitation delivery fibers imposed minimal burden and weight when integrated with our previously designed on-board detector system^10^, which eliminated the weight burden of plastic optical fibers and enabled imaging in lit conditions. This design is well suited to studying complex free behavior, such as gap crossing, as demonstrated here. Although the new components slightly increased the microscope mass compared with our previous version, appropriate counterweighting reduced the weight experienced by the mouse such that it could jump across a gap between raised platforms and move with little hindrance during extended imaging sessions (the longest here was approximately 30 min). The design of this multiplane microscope required four more optical fibers than previous single-plane microscopes, and the additional fibers have the potential to stiffen the cable bundle attached to the microscope. However, we found no substantial difference in behavioral performance between animals carrying the multiplane microscope or just the LEDs used for position tracking, or proxy microscopes with one or five optical fibers. Further, the measured characteristics of animal behavior were also similar to those measured from animals carrying a previously described miniature microscope^10^. Together, these results suggest that the effects of the combined weight of the microscope and cable bundle can be effectively mitigated by a well-designed suspension and counterweight system.

One major concern about this multiplane microscope was the increased amount of energy entering the tissue^40^. Although the total number of pulses delivered into the tissue was in the same range as our previous microscopes, the combination of both 2PE and 3PE could potentially create tissue heating and damage^48^. Using commonly described measurements of tissue health^45^, we could not detect any damage associated with excessive energy absorption throughout the five imaging planes, even after continuous imaging sessions of >30 min.

The major advantages of the multiplane microscope are its ability to visualize activity in all neurons in a population, including those firing infrequently; to unambiguously record the same neurons over prolonged periods; and to facilitate definition of neuronal subpopulations using selective expression of fluorescent markers in the recorded populations. However, neuronal activity is recorded as changes in intracellular Ca^2+^ concentration, as a proxy for firing of action potentials. Development of methods for deconvolution of the Ca^2+^ fluorescence traces into the underlying sequence of action potentials is a flourishing field^49–59^, with the algorithms providing ever-improving fidelity relative to simultaneous ground-truth recordings of neuronal electrophysiology and Ca^2+^-fluorescence^60,61^. However, because of the non-linear Ca^2+^-binding properties of calmodulin-based genetically encoded calcium indicators, it is difficult to accurately deconvolve Ca^2+^ fluorescence traces from neurons firing vigorous trains of action potentials^62^. Furthermore, the ground-truth data used for validating deconvolution methods are derived from neurons in superficial layers, and the algorithms have yet to be fully validated for neurons in deeper layers. In our analyses, we used a statistical approach to determine the onset of neuronal Ca^2+^ transients because there is a more direct relation between the Ca^2+^ fluorescence trace and underlying action potentials at these times. Additionally, deconvolution methods can be applied to Ca^2+^ fluorescence data from other microscopes, including those from the multiplane microscope.

The multiplane microscope described here enables the imaging of activity from neuronal populations located in both L5 and L2/3 during complex decision-making behaviors, during which the animals had to estimate gap distance and cross from one platform to another to receive a reward. We imaged neuronal populations located throughout the posterior parietal cortex during decision-making behaviors in mice because the correlation between behavior and neuronal activity in the upper cortical layer is well established^46,63–65^. Recording over many imaging sessions, during which animals were free to perform the crossing task at will, we established the differences between neuronal activity patterns in L2/3 and those in L5, relative to the animal’s behavioral outcome. Using this new microscope, which enables near-simultaneous recording of neuronal activity across cortical layers in animals performing decision-making tasks, together with high-resolution circuit-reconstruction techniques^24^, questions about how individual neurons located across the cortical layers transform sensory-derived inputs^25^ during free behavior can be addressed at the circuit level.

## Methods

### Animals and surgical procedures

#### Animal protocol

All animal experiments were conducted in accordance with the animal-welfare guidelines of the Max Planck Society and with animal experimentation approval granted by the Landesamt für Natur, Umwelt und Verbraucherschutz Nordrhein-Westfalen, Germany (protocol no. 84-02.04.2020.A403).

Seven male wild-type C57Bl/6 mice (*Mus musculus*) were obtained from Charles River. The mice were housed in a specific-pathogen-free temperature-controlled (21 ± 1°) and humidity-controlled (>45%) facility on a 12-h light–dark cycle, with food and water available ad libitum. Mice were group-housed until surgery and singly housed afterwards. At the start of the experiment, the mice were between 26 and 31 g (mean, 28 g).

For virus injections, we used AAV1/2-syn-jGCaMP7f based on the plasmid pGP-AAV-syn-jGCaMP7f-WPRE (plasmid no. 104488), described in ref. ^66^.

The example data presented in Figures 2, 3a–c and 4 are each derived from different animals. Data in Figure 5a–c are derived from the same animal and experimental session as that described above and shown in Figure 4.

#### Surgical procedures for fluorescent labelling of neurons with jGCaMP7f

Before surgery, all instruments, including the glass injection capillary, were sterilized by either autoclaving or heat sterilization. Animals were anesthetized with an intraperitoneal injection of a three-component anesthetic cocktail (FMX) consisting of fentanyl (50 µg kg^−1^, Hameln Pharma Plus), midazolam (5 mg kg^−1^, Hameln Pharma Plus) and xylazine (10 mg kg^−1^, WDT). Body temperature was maintained at 37–37.5 °C with a heating pad and heater controller (FHC). Animal status and depth of anesthesia was monitored approximately every 15–30 min. Anesthesia was maintained with supplementary doses of 30–80% of the above anesthetic combination, given as necessary to maintain absence of withdrawal and corneal reflexes. The animals were then placed in a stereotaxic apparatus, the hair on the scalp was removed and the skin was cleaned with 70% ethanol. The right parietal bone was exposed, and a burr hole (approximately 500 µm in diameter) was drilled at 3.6 mm posterior and 2.6 mm lateral to the bregma for experiments in the visual cortex (V1), and 2.1 mm posterior and 1.4 mm lateral to the bregma for experiments in the posterior parietal cortex (PPC). A small slit was made in the dura beneath the burr hole, and a glass injection capillary with a beveled tip containing a high-titer solution of AAV1 and AAV2 coding for jGCaMP7f was advanced into the cortex. For V1 experiments, the pipette was angled at 25° relative to horizontal and was advanced posteriorly along a trajectory parallel with the sagittal suture. Three virus injections were made, targeted to depths of 600 µm, 500 µm and 300 µm (tangential to cortical surface) and with volumes of 80 µl, 50 µl and 50 µl, respectively. The deepest of the injections was aimed 4.5 mm posterior and 2.6 mm lateral to the bregma. In the PPC experiments, the pipette was angled at 28–31° relative to horizontal and was advanced posteriorly along a trajectory parallel with the sagittal suture. Three virus injections were made, targeted to depths of approximately 450 µm, 320 µm and 200 µm (tangential to cortical surface), each with a volume of 200 µl. In all cases, there was a 5-min delay between the end of the virus injection and when we moved the pipette to the next injection location or withdrew it from the cortex. After pipette withdrawal, the craniotomy was sealed with medical silicone (KwikSil, WPI), and the skin was sutured closed using 5/0 vicryl sutures (Ethicon). Approximately 30 min before the completion of the procedure, the animals were administered buprenorphine (30 µg kg^−1^, Bayer) and carprofen (5 mg kg^−1^, Zoetis) for post-operative analgesia. After the completion of the procedure, a cocktail of antagonists to the anesthetic drugs (anti-3K) consisting of naloxone (11.2 mg kg^−1^, Ratiopharm), flumazenil (0.5 mg kg^−1^, Hikma) and atipamezole (10 mg kg^−1^, Orion Pharma), was administered.

#### Surgical procedures and imaging in freely moving animals

All surgical instruments and solutions used were autoclaved before the procedures described below. Three to five weeks after the surgery to label neurons with jGCaMP7f, animals were anesthetized using the FMX cocktail described above, and body temperature was maintained at 37–37.5 °C. Procedures for monitoring animal status and depth of anesthesia were done as described in the ‘Surgical procedures for fluorescent labeling of neurons with jGCaMP7f’ section. Anesthesia was maintained with supplementary doses of 30–80% of the FMX solution. The hair on the dorsal aspect of the skull was removed, and the skin was cleaned with 70% ethanol. A midline incision in the skin over the parietal bones was made, the skin was retracted and galea was removed to expose the parietal bones, including the site of the previous burr hole. The exposed bone was then cleaned with hydrogen peroxide solution (3% by volume in sterile saline) and thoroughly washed with sterile saline. The bone was then mechanically roughened before application of a layer of dental adhesive (Optibond). A custom-made headplate, with a central circular aperture measuring 3.3 mm in diameter, was then fixed to the skull over the Optibond layer with dental composite (Charisma). The central aperture was placed such that the burr hole from the previous surgery was located approximately centrally in the medial–lateral axis, near the anterior edge of the aperture. The skin incision was then closed firmly around the headplate using 5/0 vicryl sutures (Ethicon). A circular craniotomy with a diameter of roughly 3 mm was then opened in the center of the headplate aperture, including at the anterior margin of the site of the previous craniotomy. The dura was removed, and the cranial window was closed using a pre-formed plug and coverslip (circular, 5 mm diameter, 100 µm thickness, CS-5R-0, Warner Instruments; plug 300-µm-tall glass cylinder fixed to the coverslip using UV curing optical glue (Norland Optical adhesive No. 68, Edmund Optics)), which was secured to the base of the headplate using transparent biocompatible silicone (KwikSill). The plug was designed to approximately match the gap between the surface on which the coverslip rested and the bottom of the bone, including the thickness of the headplate and the adhesives used to fix it to the skull. The cranial window was protected using a plastic 3D-printed cap, which was secured in place on the headplate using a fastening screw. Postoperative analgesia and anesthetic antagonist solution composition, dose and administration were the same as described above in ‘Surgical procedures for fluorescent labeling of neurons with jGCaMP7f.’

The headplate consisted of a printed component forming the walls of the head-plate chamber and the stalk to hold it, which was printed in a biocompatible, glass-filled resin (Temporary CB and Form 3 printer, Formlabs), and a 80-µm-thick steel ring (6.9 mm diameter with a central aperture measuring 3.3 mm in diameter). The two head-plate components were then fixed together using dental composite (Charisma, Kulzer).

#### Placement of the multiplane microscope

Two to four weeks after the procedure to install the cranial window, the animals were anesthetized with isoflurane (1.25–2.5%, Baxter), delivered in air at a flow rate of 1.5 L min^−1^, and were then transferred to the setup for mounting the miniature microscope, which was mounted on a navigation stage for locating an appropriate position for imaging within the cranial window. The navigation stage consisted of a micromanipulator (MP-285, Sutter Instruments), to which the microscope was mounted using a custom-made mount. The mount included two angular kinetic mounts (cat. nos. GN05/M and GN1/M, Thor Labs), allowing the tilt to be adjusted with respect to the coverslip and cortical surface. Once a target population of neurons had been located, the intermediate attachment plate (already mounted to the miniature microscope) was attached to the headplate using dental composite (Charisma Flow, Kulzer). The microscope was then removed, a plastic 3D-printed protective cap was attached using the same mounting mechanism used to secure the microscope in place, and the animal was allowed to recover.

#### Histology

At the end of the experiment, animals were deeply anesthetized with ketamine (75 mg kg^−1^) and xylazine (10 mg kg^−1^), and perfused transcardially with 0.1 M phosphate buffer (PB) followed by 4% formaldehyde solution (Roti-Histofix, Carl Roth). The brain was then removed, post-fixed at 4 °C overnight in the same formaldehyde solution and then transferred to 0.1 M PB. Sections of 100 µm thickness were then sectioned using a vibrating microtome (Leica VT1000S, Wetzlar) and mounted in fluoroshield (Sigma). Images were acquired on an inverted microscope (Nikon Ts2R-FL).

#### Training gap-crossing task

To introduce the animals to the task and reward, they were initially placed in a home ‘jumping’ cage (dimensions 55 × 35.5 × 30 cm, length × width × height) containing both launch and reward platforms (dimensions below in ‘Freely moving sessions’) with water and/or sweetened cereal pieces (Froot Loops, Kellog’s) as rewards on top of the reward platform. Launch and reward platforms were initially positioned adjacent to one another so that the animals could climb freely between them.

Rewards on the reward platform were replenished regularly to promote frequent exploration and cycling. Once mice were comfortably cycling between blocks (roughly 1–2 days training) the distance separating the launch and reward blocks was increased incrementally until the animals had to jump to obtain the reward. These distances continued to be increased, up to a maximum of 20 cm, or until an individual mouse refused to cross more often than it crossed successfully. During periods of water restriction, daily water allowance was delivered either on the reward platform or during discrete 10- to 30-min training sessions, in which each successful cross was accompanied by a water reward of approximately 0.1 ml and/or a sweetened cereal piece. The daily water allowance was determined separately for each mouse, set at 50% of their ad libitum water consumption by design, with a minimum of 30%. Finally, to habituate the mice to both the experimental arena and the experimenter, each animal underwent at least three 30-min acclimation sessions in the laboratory with the microscope and all supporting equipment, using the same platform configuration and arena used in the recording sessions. The mice were allowed to cross freely, and received a reward after each cross.

#### Freely moving sessions

Here, we define an experiment as a group of recording and imaging sessions with a single animal. Freely moving sessions were conducted from 9–11 weeks after the cranial window was opened. At the start of each recording session, the animal was taken from its home cage, its head was gently restrained by holding the handle on the back of the headplate, and the microscope was placed onto the intermediate attachment plate and secured in place with a holding screw. Following this, the animal was placed in an open rectangular arena measuring 30 × 50 cm^2^ for experiments in V1, or a walled rectangular arena measuring 32 × 50 cm^2^ with 15-cm-tall walls for PPC experiments. In these arenas, we placed two movable platforms, the reward platform measuring 10 × 10 × 20.5 cm^3^ and the launch platform measuring 10 × 10 × 12.5 cm^3^, with an attached short step measuring 10 × 5 × 6.5 cm (all measurements, length × width × height). The mice in the PPC experiments were water-restricted, as described above. Mice that consumed <30% of their ad libitum intake in the behavioral arena were given supplementary water making up the difference between their consumption in the arena and the 30% threshold at the end of the session in their home cage. They were further encouraged to cross between platforms using small pieces of breakfast cereal. At the end of each recording session, the animal was gently removed from the arena, the screw on the microscope mounting plate was unfastened and the microscope removed, the protective cap was put in place and the animal was returned to its home cage.

#### Behavior comparison

To measure the effect of the microscope and associated fiber bundle on measurable parameters of mouse behavior and movement, we first created a proxy microscope, weight-matched to the multiplane microscope described above, onto which either a fiber bundle including five optical fibers or one with a single fiber could be attached. We also made a head mount consisting only of the LEDs used for position tracking. We then made 10-min continuous recordings of the animals’ position in the open arena as they sequentially carried each type of equipment: the proxy microscopes, the tracking LEDs and the real multiplane microscope. Recordings were made over multiple days, with a maximum of two recordings for a single animal on any day. The sequence in which each animal carried the four test devices was randomly varied between each animal.

### Behavioral setup and animal tracking

#### Environment light

The track environment was homogeneously illuminated with six 24 V RGBW LED strips, each 125 cm in length with 910 lm m^−1^, alongside eight 12 V white LED strips of the same length, delivering 700 lm m^−1^ (both LED strips from PowerLED), arranged equidistantly in a patch of 125 × 60 cm^2^ at a distance of 150 cm above the track.

The strips were switched on and off in synchrony with the line signal of the microscope using a custom circuit based on TIP120 Darlington transistors.

#### Tracking animal position

Animal position in the arena was tracked using a system of overhead cameras and position-tracking LEDs mounted onto the microscope previously described in ref. ^67^. To mount the LEDs onto the microscope, three struts were mounted onto the microscope body, with three infrared (IR) LEDs (940 nm, cat. no. SFH 4053, Osram) mounted uniquely per strut. Images of the IR-LEDs were acquired at 200 Hz by four calibrated and synchronized digital cameras (cat. no. acA1300-200um, Basler) mounted above the arena. The cameras were equipped with 4.4–11 mm, F1.6 objective lenses (cat. no. LMVZ4411 1/1.8” F1.6/4.4–11 mm, Kowa) to facilitate coverage of the arena and IR bandpass filters (cat. no. BN 940-43, Midwest Optical Systems) to facilitate tracking of the LEDs only. The exposure active signals (a TTL signal set high while the camera is capturing an image) from the overhead tracking cameras as well as the frame synchronization signal from the miniature microscope were fed into an analog-digital converter (cat. no. Power 1401, Cambridge Electronic Design) and recorded with Spike2 software for synchronization of behavioral tracking and multiphoton imaging data. Animal position and head orientation were tracked as described in ref. ^67^, with the exception of two datasets in which the positions of the IR LEDs in the camera images were tracked using DeepLabCut^68,69^. The detected positions were then used for determination of animal pose and position, as described in ref. ^67^. All cameras were calibrated using the calibration procedure described in ref. ^70^.

### Hardware

#### Miniature microscope optical configuration and production of the prototype

The optical system of the multiplane microscope, from MEMS scanner to objective and detection system, is largely similar to the previously described miniature multiphoton microscope^10^, with the following components. The scanner is a 2.0-mm-diameter MEMS scanner (cat. no. A7M20.1-2000AU-LCC20-C2TP, Mirrorcle), featuring ±5 mechanical degrees of scanning range and 1.7 kHz scanning frequency. All lenses and mirrors off-the-shelf used were custom-adjusted by centering and thinning down (Throl Optics) to fit mechanical specifications of the microscope (Supplementary Fig. 1). The scan and tube lens consists of a custom doublet lens and a plano-convex (PCX) lens with an effective focal length (EFL) of 12 mm (3 mm diameter, (cat. no. 67-444, NIR II, Edmund Optics). A dichroic mirror (cat. no. T875spxrxt, AHF Analysentechnik) was used to separate the excitation beam path and the emitted fluorescence. A condenser lens with an EFL of 5 mm was used (Throl Optics) followed by an infrared filter (cat. no. G380227032, Qioptiq Photonics). A dichroic long-pass mirror (490 nm, cat. no. DMLP490R, Thorlabs) separated emitted light into two channels, one detecting light emitted from third harmonic generation (THG, blue) and one for GCaMP7f (green). We used SiPM multi-pixel photon counters (cat. no. S13360-1375PE, Hamamatsu) as on-board detectors mounted on the microscope.

The high-speed version of the microscope was identical to the previously described miniature multiphoton microscope^10^. In brief, the scan lens consists of a custom doublet lens and a plano-convex (PCX) lens with an effective focal length (EFL) of 12 mm (3 mm diameter, cat. no. 67-444, NIR II, Edmund Optics), and the tube lens was the same custom doublet lens alone. The same custom objective lens was used as that for the large-field-of-view version. The full prescription of the high-speed version is available in the previous study^10^. The scanner used for fast scanning (cat. no. A7M10.2-1000AL -LCC20-C2TP, Mirrorcle) featured ±4.5 mechanical degrees of scanning range and 4.0 kHz scanning frequency.

The optical system between the end of the optical fiber and the MEMS scanner for the 3PE path consisted of a 2-mm-diameter, 5-mm-EFL aspheric collimation lens (cat. no. 354430-C, Thorlabs) and two dichroic mirrors (cat. no. DMSP1180R, Thorlabs). For the 2PE path, it consisted of a 2-mm-diameter, 4-mm-EFL achromatic lens (cat. no. 84-128, NIR II, Edmund Optics) and a stack of µT lenses (Packaged TLens Silver IRSM – PIF.15.P01, PoLight ASA) for adjustment of the axial offset between the 2PE image planes and 3PE imaging plane after the objective lens. An additional single µT lens common for both 2PE and 3PE was inserted before the MEMS scanner for fine axial position adjustment. The full optical system of the miniature microscope is shown in Figure 1a and Supplementary Figures 1 and 2, and its various parameters were optimized using OpticStudio 14.2 (Zemax Europe).

The production of the prototype has been described in detail in previous work^10^. In brief, the structure of the microscope body, implants and lens-mounts, including the objective, were designed with Inventor (Autodesk). The parts for the microscope body were printed in black resin V4 (Formlabs) on a 3D printer (Form3, Formlabs). All the optics and the objective were supplied by Throl Optics. The four fibers for the 2PE path (cat. no. HC-920-PM, NKT Photonics) were jointly mounted in a 3D printed ferrule (Fig. 1c,d) with a diameter of 1.25 mm, which was printed in yellow resin (HTL -Yellow Resin, BMF Precision) on a high-resolution stereo-lithography printer (microArch S230, BMF Precision). The ferrule was then mounted in a corresponding structure in the microscope (Fig. 1a,c,d). The fibers were cleaved, and the coating was removed with a razor blade over 10–15 mm, then manually inserted in the ferrule and glued inside with UV-curing glue (cat. no. NOA068, Thorlabs). A manual fine-tuning of each fiber’s axial position was necessary to achieve the 80-µm offset between the corresponding imaging planes before the final gluing of the fibers. For the 3PE path, the fiber was mounted in a similar 3D-printed ferrule but designed with a single fiber bore (Fig. 1d and Supplementary Fig, 2), with the ferrule then mounted into its corresponding structure on the microscope (Fig. 1a,d). Fiber cleaving and mounting in the ferrule were as described above for the 2PE path.

#### Synchronous multimodality excitation laser setup

For excitation, we used an optical parametric chirped pulse amplifier (OPCPA), pumped by a Ytterbium fiber laser (white dwarf dual, Class 5 photonics) that produced 1,300-nm pulses with a maximum average power of 4 W (2 µJ at 2 MHz; 3P excitation beam) as well as 960-nm pulses with a maximum average power of 1 W (0.5 µJ at 2 MHz: 2P excitation beam). We used a half-wave plate (cat. no. AHWP05M-1600, Thorlabs) mounted on a rotation mount with a resonant motor (cat. no. ELL14, Thorlabs) with a polarization beam splitter (cat. no. PBS104, Thorlabs) to control beam intensity for the 3P beam and a Pockels’ cell (cat. no. 350-80LA, POLYTEC) for the 2P beam. In the 3PE path, we compensated for dispersion introduced by the fiber using the two-prism sequence with additional bulk silicon, described previously^9^. In brief, to compensate for third-order dispersion (TOD), a double-path two-prism sequence compressor with Brewster-angle silicon prisms at 32° apex angle (cat. no. 4155T724, Korth Kristalle) was used. The custom-designed second prism had a larger 56-mm base to increase the compensation range. Additional anomalous group-velocity dispersion (GVD) generated by the prism sequence was compensated using bulk silicon. To compensate for the dispersion of the 1.5 m of the hollow-core fiber^9^ used in this study, we used an inter-prism distance of 35 cm with an additional 10 cm of bulk silicon. For 2PE, the GVD was minimized by adding 5 mm ZnSe slabs at Brewster angle (cat. no. WG71050, Thorlabs), altogether a total path of 16 cm, to compensate for approximately 1.75 m of fiber (HC-920-PM, NKT Photonics). The beams were coupled into their respective fibers using a range of achromatic coupling lenses with EFLs from 7.5 mm to 20 mm (AC050-XXX-C-ML, Thorlabs), with the optimal lens chosen for maximum coupling efficiency.

To create a timing structure for the laser pulses that allows multiplexed excitation of fluorophores, we used cascaded pairs of half-waveplates (cat. no. AHWP10M-980, Thorlabs) and polarizing beam splitters (cat. no. PBS105, Thorlabs), each pair splitting laser pulses in two and allowing continuous control of the power splitting ratio. After each splitting step, the reflected beam was then coupled into a fiber, with an approximately 8-ns delay added to the transmitted beam to achieve the required time separation between successive pulses fitting the designed demultiplexing strategy. Each fiber’s symmetry axis was aligned to the axis of the linear polarization of each incident beam for optimal transmission and temporal profile. For the last fiber, the transmitted beam of the third beam-splitter was used. Each pulse propagates through its designated fiber, exciting a volume of fluorophore at the corresponding plane (Fig. 1a–d and Supplementary Fig. 2). The splitting ratio was manually adjusted for each animal to achieve similar levels of excitation for each channel. All four 2PE channels were then jointly modulated with the common Pockels cell for power adjustment, flyback and fill fraction blanking. The achieved time structure of the multiplexed excitation beam was verified using a fast photo-diode (cat. no. DET10A2, Thorlabs).

#### Fluorescence detection, control electronics and software

Scanimage software and a high-speed analog-to-digital and digital-to-analog converter board (cat. no. ScanImage 2023.1.0 premium, hsViDAQ, MBF Bioscience) were used to control the microscope and acquire the data. The MEMS scanner was operated in the resonance mode in the fast axis at 1.7 kHz, providing 3,400 lines per second using bi-directional scanning and 3 ms flyback. This, combined with a 2-MHz excitation laser pulse rate and 80% of imaging fill fraction, resulted in a resolution of about 410 pixels × 340 lines at a frame rate of 9.73 Hz. The field of view during data collection measured 600 × 450 µm^2^. The driving signals from Scanimage software were sent to the MEMS driver (cat. no. BDQ_PicoAmp_4.6, Mirrorcle). A high-bandwidth preamplifier was custom-designed on the basis of publicly available schematics from the evaluation board of the SiPM (cat. no. S13360-1375PE, Hamamatsu). Gating of acquisition to separate the sequential pulse sequence was achieved by adjusting the FPGA parameters using ScanImage 5 built-in features.

#### Counterweight system

The counterweight system (Supplementary Fig. 9a) consisted of a lightweight, 3D-printed, 20-cm-long suspension arm that was suspended approximately 130 cm above the level of the arena floor. One end of the suspension arm was attached to the microscope’s cable bundle approximately 60 cm from the microscope using an approximately 80-cm length of fishing line (0.355 mm diameter, Select Fluorocarbon, Climax Fishing Lines). The other end had a magnetic attachment for attaching counterweights, with the weight adjusted until the microscope was counterbalanced to levitate 2–3 cm above the arena floor. The pivot point on the suspension arm, through which it was attached to an overlying structure, was 7 cm from the end with the counterweights.

### Data analysis

Raw imaging data were first preprocessed to minimize scan-pattern distortions, brain motion and cross-talk between gated channels as described below. The photon-count-related signal structure of the SiPM’s used on the microscope allowed all data pre-processing steps to employ photon-flux as the measurement unit, which was determined in the initial photon calibration step. Expected emission photon counts per pixel are on the order of 1 to 10^71^.

#### Photon calibration

The pulse-height distribution of the SiPMs exhibited a prominent peak structure related to *n*-photon detection events. To infer the scaling factor from the ADC signal level to photon-count we first characterized the Gaussian peak and width parameters of the noise floor. We then identified the 1- to *n*-photon peaks using a moving-window correlation with a Gaussian with parameters of the noise floor. The pulse-height to *n*-photon scaling factor was then defined as the average of the difference between the 1- to *n*-photon peaks, and the 0-photon offset was inferred by subtracting the scaling factor from the 1-photon offset. These parameters were then applied to the image sequence using floating-point arithmetic.

#### Cross-talk de-mixing

The primary sources of cross-talk were first the time course of fluorescent decay and the analog high-frequency behavior of the SiPM detector, cables and pre-amplifier, both having an impact on the scale of approximately 10 ns, and second the after-pulsing of the SiPMs (spurious pulses elicited by the SiPMs after a photon or dark capture event identical in amplitude and shape to the original pulses), which occurs with a delay of ten to several hundred nanoseconds. As the excitation delay between channels is 8 ns, both sources can result in spurious pixel intensities in trailing channels (time range approximately 32 ns), but not in next pixel (time range 500 ns) or next frame (time range 100 ms). The net result is the appearance in the trailing channels image of dim replicas of labeled structures from the leading channel at the same locations as in the leading channel.

To remove the cross-talk between leading and trailing channels, we determined the cross-talk coefficients *C*_*mn*_ for each pair of

$$C=\left\{{C}_{{mn}}\right\}$$

leading and trailing channel denoted as *n* and *m*, respectively, which contained only non-zero coefficients for *m* > *n*, so that each channel-wise image series *I*_*n*_ (each image series having identical width, height and frame count) could be de-mixed using

$${I}_{m}^{{\prime} }={I}_{m}-\mathop{\sum }\limits_{n\geqslant 0}{C}_{{mn}}{I}_{n}$$

To determine the cross-talk coefficient for each relevant channel pair, we first calculated the selected-pixel mean correlation coefficient (spMCC) to estimate the extent of the cross-talk between the leading and trailing channels. To minimize the impact of unlabeled regions in the image, we identified all non-saturated pixels (pixels with a maximum photon flux < 30) for which the s.d. of the low-pass-filtered data (Gaussian low-pass, *σ* ≈ 2.5) was greater than 0.5. We then calculated the spMCC as the average of the pixel-wise Pearson’s correlation coefficients between the leading and trailing channels for the selected pixels. The cross-talk coefficient for each relevant channel pair was then determined by minimizing the spMCC.

#### Motion correction and scan-pattern correction

For optimal motion-correction performance, the physical pixel size must be constant over the image. However, this is not the case when using a sinusoidal drive signal to the MEMS scanner. To address this, we used non-linear resampling over the fast axis. To avoid aliasing during that process, we first upsampled the fast axis tenfold, then non-linearly downsampled it for spatial uniformity using the resample Matlab function with default parameters for low-pass filtering. For convenience, we chose a final pixel resolution of 512 × 512 as a balance between more pixels for higher-resolution ROI definition and precision of motion correction while limiting the increase in data size and processing time. Motion correction was performed using a custom Matlab port of the image registration component of suite2p^72^, involving a displacement estimation using phase-correlation and the subsequent rigid frame shift. To increase robustness under low-photon-count conditions (in which dark counts interfered with the phase-correlation), the image series was filtered in time (Gaussian low-pass, *σ* ≈ 1.5).

#### Selection of regions of interest in imaging data

ROIs were selected manually in the current datasets, with boundaries around individual neuronal somata or dendrites being defined around the selected structures in the average image of the green fluorescence channel. ROIs were included in subsequent analysis regardless of whether obvious fluorescence transients were present. No additional selection criteria were used.

#### Demonstration of automated ROI detection using Suite2P

To verify that the data produced by the multiplane microscope is suitable for automated analysis pipeline, we used a matlab version of suite2p^72^. We visually optimized the parameters of detection. The optimized parameters were: cell diameter, 12 pixels; spatial smoothing, 1 pixel; SVD components number, 300. The unfiltered result often produced neurons broken into several components, usually featuring a main component and a few bad copies around it. After the segmentation using suite2p, the extracted ROIs were additionally filtered to remove bad copies using a distance and correlation criteria. We used a moving average window of 20 frames to remove the high-frequency noise impact on the correlation metric. We then computed the pair-wise distance between the centers of all ROIs and the correlation matrix (between each pair of ROIs). We defined a distance threshold (*D*_th_) of 1.5 times the median ROI diameter and a correlation threshold (*C*_th_) of the average correlation between two ROIs plus 3 times the s.d. of this correlation. In each neighborhood, we removed ROIs with a correlation divided by distance larger than *C*_th_/*D*_th_ with another ROI. We consistently maintained a brighter ROI on average to remove bad copies but keep the main component. This joint approach led to a stable automated segmentation. An example of such segmentation is shown on Supplementary Figure 7.

#### Extraction of fluorescence signal

The fluorescence signal was calculated for ROIs by averaging pixel values per frame and ROI. Alternatively, to yield photon flux, pixel values were summed up.

#### Calculation of Δ*F*/*F*_0_

The baseline fluorescence (*F*_0_) was defined as the mean of the lowest 20% of fluorescence values within a window of maximally 200 s around the current frame (if not truncated by the beginning or end of the file) after applying a Gaussian filter with a s.d. of 282 ms.

#### Point spread function

The point spread function was measured using a sample of 500 nm fluorescent beads (cat. no. F8813, Molecular Probes) suspended in an agarose gel, using image stacks with a 1-µm *z*-step (average measured optical resolution, axial 22.2 ± 1.8 µm, lateral 1.45 ± 0.11 µm for 2PE channels and axial 14.8 ± 1.1 µm, lateral 1.30 ± 0.03 µm; all mean ± s.d.).

#### Side-view imaging of fluorescent agarose gel

To observe the relative position of the laser spots corresponding to each excitation beam, we placed the microscope above a fluorescent agarose gel-filled glass cuvette. A side-view camera with a high-resolution objective lens (cat. no. MVL25TM23, Thorlabs) was used to record the fluorescence resulting from the excitation of the fluorophore from the side (Supplementary Video 1).

#### Ground-truth data for de-mixing algorithm

To provide ground-truth data for the multiplexed channels de-mixing algorithm, we acquired sequential images along the axial dimension of 10 µm fluorescent beads (cat. no. F8836, Molecular Probes) suspended in an agarose gel. The imaging was first performed with all excitation beams operating, then the same volume was imaged sequentially using only one fiber at each acquisition. For the latter acquisition, all unused laser beams were physically blocked.

#### Quantification of de-mixing algorithm performance

To quantify the performance of the de-mixing algorithm, we first rescaled the ground-truth data described above from all channels to have the noise-floor centered at 0 and the maximum level normalized to 1, to exclude the impact of relative brightness of each channel, before computing the cross-talk. We then used the scaled ground-truth images from the first four channels (leading channels) to produce ROIs around the beads using the imbinarize Matlab function, generating one set of ROIs for each leading channel. For each leading channel, we applied the ROIs to all trailing channels and summed the absolute values of these signal levels. Absolute values were used to capture negative cross-talk between channels in the cross-talk metric. We summed these numbers obtained from each leading channel for a given condition (ground-truth, de-mixed and raw data). For the de-mixed and raw-data condition, this number is designed to contain all the additional signal in all channels that is generated owing to cross-talk. It will also contain noise and signal from objects from trailing channels that are coincidentally located in the ROIs detected on the leading channels. We therefore computed the same metric on the ground-truth data to evaluate the expected level of this metric without cross-talk. In all three conditions, the data contain exactly the same sample, and the same channels have been registered from raw or de-mixed condition to ground-truth reference channels to have objects located in the same parts of the image.

#### Detection of frames corrupted by chewing on behavioral reward

Following a successful gap cross onto the reward position, the animals were given a crispy Froot Loop as a reward. Chewing on the Froot Loop corrupted a subset of imaging frames beyond recovery, probably by generating high-frequency resonance in the MEMS scanner. The affected frames made up <5% of the data and occurred exclusively following the reward delivery, while the animal was chewing. To detect the affected frames, we calculated the frame-wise fast correlation (FFC). To calculate the FFC, we first calculated the Pearson correlation between each frame and the template produced in the process of motion correction. Then, to remove the influence of slow image drift, we low-pass filtered the FFC trace (movmedian Matlab function with width of 100 frames) and subtracted this from the original FFC trace to generate the final FFC metric. We next fitted a two-component Gaussian mixture model to the distribution of FFC values using the Matlab function fitgmdist. The component with the lower average FFC was manually confirmed to correspond well to the corrupted frames. Corrupted frames were then defined as all frames for which the FFC was lower than the lower component mean FFC plus two s.d. of the lower component.

When calculating neuronal or ROI fluorescence traces, the corrupted frames were excluded with the trace interpolated between the neighboring non-corrupted frames.

#### Onset time of neurons in sequences of neuronal activity in PPC

To characterize the sequential activation of neuronal populations in PPC, we used a triggered average of fluorescence trace of each neuron (segmented region of interest) on behavioral events. First, the events were manually analyzed using the visible light cameras data during offline analysis. Crosses from all platforms to the floor or to other platforms, reward events and refusals were labeled. The imaging data were averaged in a window from −20 s to +30 s around each class of behavioral events. For further analysis, only data for the ‘EAT’ event was presented in the current work. For comparison, the same number of randomly selected events was used to generate shuffled ‘RANDOM’ data. The first 10 s of each such average was used as a baseline to assess the variability of the neuronal activity more than 10 s before the behavior. To do that, we calculated the average and s.d. of the trace for each neuron, then used these values to compute a *z*-score for the resting 40 s of data (interval [−10, 30] around the detected behavior). We then applied a threshold on the data that had a *z*-score above 3. The first such event with a duration larger than 0.5 s for each neuron occurring in the given interval was then used as the onset of the corresponding neuron in the sequence. Neurons with no detected events were not attributed any onset time and were not considered for further sequence analysis. Raster plots with one averaged and binarized neuronal per row and ordered on the *y* axis from earliest (top) to latest (bottom) onset time are shown in Figure 5d and the corresponding shuffled version in Figure 5e. The detected onset times for each neuron were labeled in red.

To build the cumulative probability function from all imaging sessions from all animals, we combined the onsets of all sequence cells, as they were detected on the same time interval around the cross, then sorted them in ascending order and used these times as *x* axis data, with the cumulative percentage on the *y* axis. The 95% confidence interval was produced using the Matlab function ecdf. The histogram data in Figure 5g represent the time-binned aggregated from the onset data of all imaging sessions. The error bars were calculated under the assumption of a locally uniform neuron onset density over time, resulting in a Poisson distribution of the onsets for each given bin, giving the s.d. of the distribution of the square root of the mean.

To confirm the significance of the measured differences between behaviorally aligned trigger averages and the shuffled counterparts, for each considered time bin (data were combined with a bin-width of 5 s), we computed the differences of neighboring onset times using the onset data pulled from all imaging sessions. We computed the distribution of these differences for each time bin in both the behaviorally aligned data and the shuffled data. We assessed the significance of the observed differences using the Wilcoxon rank-sum test (Matlab function ranksum) between the behaviorally aligned distributions with the shuffled ones, and the statistical significance threshold was set at *P* < 0.05. Time bins that were significantly different were marked with an asterisk (Supplementary Fig. 12 and Supplementary Table 4).

#### Threshold-based determination of activity and inactivity segments

To segregate recorded neuronal signals into periods in which neurons were active and inactive, we adapted a previously described Ca^2+^-transient thresholding technique that uses confidence intervals on the duration and intensity of the negative transients in a DFF_0_ trace to establish an appropriate threshold for defining periods of activity^46^. We first defined a baseline mean and s.d., for each neuron or structure, using the mean and s.d. of the smallest 20% of the Δ*F*/*F*_0_ data and used these quantities to convert the data for each neuron or structure to a *z*-score. Periods of activity were then defined as those lasting at least 1 s, during which the *z*-score exceeded 5 or fell below −5. Periods of inactivity were defined as the complement of the activity periods for each neuron or structure. For this procedure, we smoothed the Δ*F*/*F*_0_ data using a moving average over a five-frame window.

#### Analysis of influence of sustained 2PE and 3PE on indicators of neuronal health

Previous studies of the effects of sustained fluorescence imaging using multiphoton excitation sources have found that long-term illumination is correlated with an increase in neuronal baseline fluorescence, as well as an increase in the time constant of the decay of Ca^2+^-transients^44,45^.

To quantify changes in baseline neuronal fluorescence indicative of neuronal damage, but which were independent of global image brightness variations, we first calculated neuronal normalized raw fluorescence for each neuron. To do this, we first calculated global background fluorescence frame-wise as the mean raw fluorescence signal over all pixels that were not contained in neuronal ROIs and subtracting this frame-wise from the neuronal ROI raw fluorescence average. We then calculated, for each neuron, the delta baseline (inactive) neuronal fluorescence as the difference between the mean normalized neuronal fluorescence for all segments defined as inactive in the first and last 20% of the recordings. For this analysis, the raw data were smoothed using a moving average over a five-frame window.

For the comparison of the decay time constants of Ca^2+^-transients, we first identified transients with approximately comparable characteristics. The selection criteria for this analysis were (1) selected transients were isolated events with the previous preceding transient returning to baseline (inactive as defined above) at least 5 s before the onset of the transient of interest, and the next following transient starting at least 5 s after the transient of interest ended, (2) amplitudes between 30% and 80% Δ*F*/*F*_0_, (3) transient baseline fluorescence, defined here as the mean fluorescence in a 1-s window before the start of the transient, between −20 and 20% Δ*F*/*F*_0_ and (4) a transient rise time, the time from start to peak of the transient, of less than 2 s. For the qualifying transients, we next fitted the decay phase of the transient, defined here as the data segment from the transient peak to the end of the transient (time at which the transient returned to inactive as defined above), with a single exponential of the form *ae*^−*bx*^, using MATLAB’s fit function. To ensure comparable and reasonable fits to the data, we selected transients for which the *R*-squared (coefficient of determination) for the fit was greater than 0.9, and for the qualifying transients calculated the decay time constant (*τ*) as −1/*b*. Finally, we compared the average *τ* for qualifying transients in the first and last 20% of each recording for each neuron. For this analysis, we smoothed the Δ*F*/*F*_0_ data using a moving average over a ten-frame window.

#### Neuronal signal-to-noise ratio and Ca^2+^-transient distributions

To calculate the neuronal signal-to-noise ratio, we used periods for each neuron defined as inactive as described in ‘Threshold-based determination of activity and inactivity segments.’ We then defined the signal-to-noise ratio as the mean fluorescence signal divided by the s.d. of the fluorescence signal in the inactive segments.

To compare Ca^2+^-transient amplitude distributions, we calculated the Ca^2+^-transient amplitudes as the maximum Δ*F*/*F*_0_ fluorescence during the active period, subtracting the baseline segment, defined here as the mean fluorescence in a 1-s window before the start of the transient. For this analysis, the Δ*F*/*F*_0_ data were smoothed using a moving average over a ten-frame window.

#### Analysis of behavioral comparison data

We first set a unified duration over which the behavioral parameters were calculated at 568 s, which corresponded to the time over which behavioral tracking was possible for the dataset for the shortest valid recording time. We then calculated the mean animal velocity, mean velocity while running, fraction of time spent running and total path length while running for the first 568 s of the recording for each dataset from each animal. ‘Running’ was defined as the animal having a velocity greater than 5 cm s^−1^. Azimuth and elevation head accelerations have been quantified during the same 568 s, at all times, using 1-s averaging of absolute accelerations for comparing distributions and 60-s averaging for comparing trends during the 10-min behavioral assay.

### Reporting summary

Further information on research design is available in the Nature Portfolio Reporting Summary linked to this article.

## Online content

Any methods, additional references, Nature Portfolio reporting summaries, source data, extended data, supplementary information, acknowledgements, peer review information; details of author contributions and competing interests; and statements of data and code availability are available at 10.1038/s41592-026-03125-7.

## Supplementary information

Supplementary Information Supplementary Figures 1–12 and Supplementary Tables 1–4.

Reporting Summary

Supplementary Video 1 **Side-view of excitation pattern of the multiplane microscope**. Initial segment shows all 5 excitation spots produced by the 3PE fiber and the 4 2PE fibers. Then beams are manually closed one-by-one in the laser setup before the fiber-launches, starting by the 4 2PE beams and the last (deepest) is the 3PE beam. Then laser optical paths are opened again and ETLs are actuated through their complete adjustment range, first the 4-element stack adjusting the axial offset between the 4 2PE spots and the 3PE, then the common 1-element ETL for fine-tuning of all imaging planes simultaneously. Video has been sped-up to shorten the video, as each manual operation was taking 30 s to a minute. Each frame of the video had a long exposure time of ~2 s to enhance the visibility of the excitation spots. The imaging medium is a 1 mM deionized water solution of sulforhodamine. The multiplane microscope is above the sample, objective lens being immersed in the same solution. The video is an illustration of the spatial relationship of various excitation channels of the multiplane microscope, for measurement of point-spread-function see Suppl. Figure 3.

Supplementary Video 2 **A summary of neural activity imaging over an imaging session in mouse V1 cortical region**. Example video showing all imaging planes from most superficial to deepest (upper row of panels, left to right), all planes in depth sequence (lower left), a visible-light view of the animal on the arena (lower center) and example Ca2 + -transients from neurons in each imaging plane (lower right). Color-coding of Ca2 + -transient traces matches the outlines on the image planes. Vidoe shown approx. 10× real speed.

## Data Availability

The imaging data split into individual planes as well as tracked behavioral data are available in an Edmond (Max Planck Society) repository (10.17617/3.NW9DW0). The full raw data will be available on request due to large volumes.

## Code availability

The code used to pre-process data, including de-mixing of imaging channels is available in the following Edmond (Max Planck Society) repository (10.17617/3.NW9DW0).
