# Supplementary material for: Simultaneous two- and three-photon multiplane imaging across cortical layers in freely moving mice
Source: Nat Methods. 2026 Jun 9;23(7):1437–46. doi: 10.1038/s41592-026-03125-7 (PMC13345954; doi:10.1038/s41592-026-03125-7)
Supplement: Supplementary file 1 — Supplementary Figures 1–12 and Supplementary Tables 1–4. [file 41592_2026_3125_MOESM1_ESM.pdf]

nature methods

Supplementary information

https://doi.org/10.1038/s41592-026-03125-7

Simultaneous two- and three-photon multiplane imaging across cortical layers in freely moving mice

In the format provided by the authors and unedited

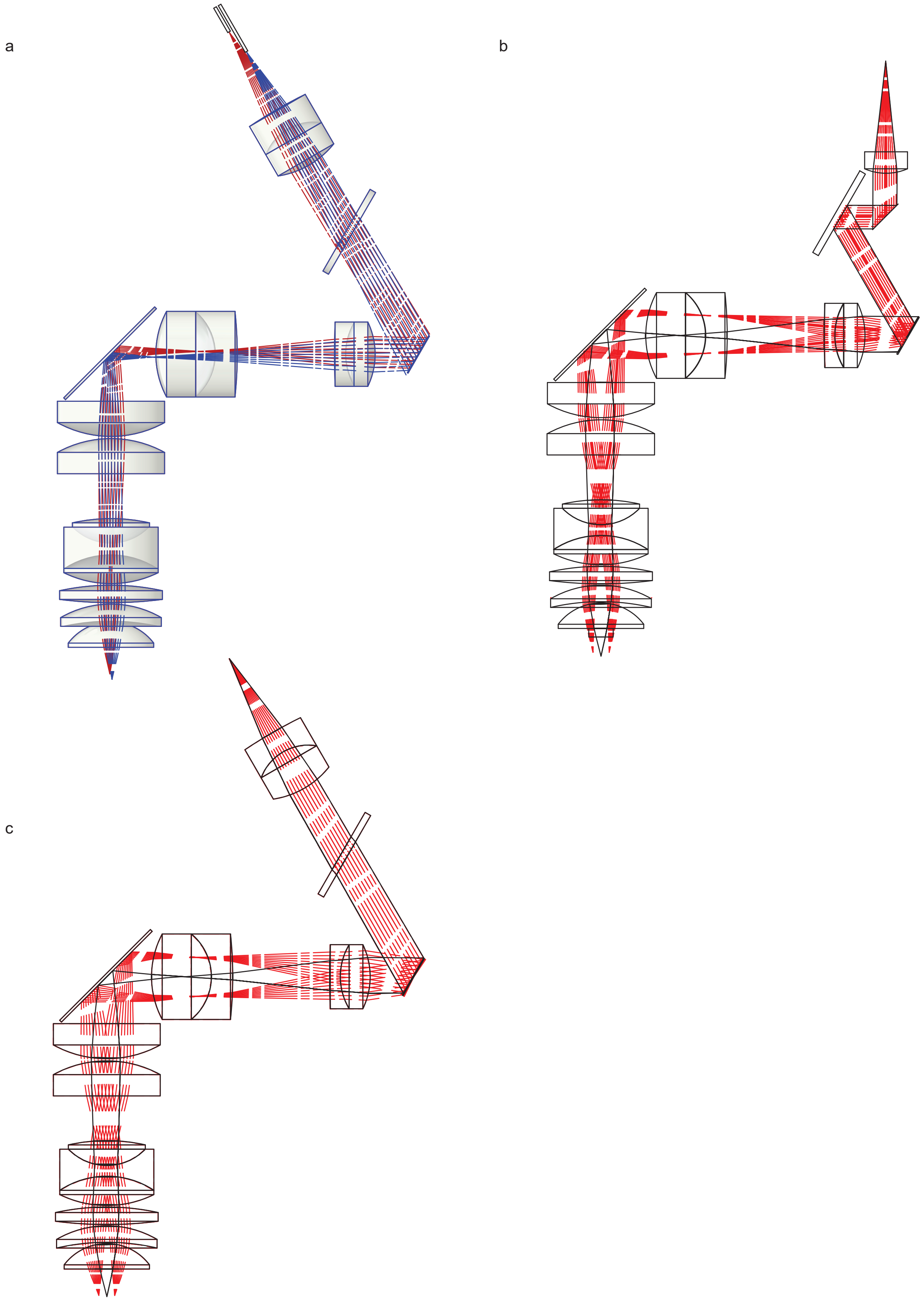

Supplementary Figure 1. Zemax model for multimodality multiplane miniature microscope. a, scaled ray tracing from Zemax model featuring extreme positions of fibers to reach >250 µm axial offsets between imaging planes and corresponding lateral distance of 300 µm between fibers for unclipped beam propagation. b, scaled ray tracing from Zemax model with optical path for three-photon excitation, 1300 nm with MEMS scanner in the center position (black) and extreme field-of-view positions (red). c, scaled ray tracing from Zemax model with optical path for two-photon excitation, 960 nm with MEMS scanner in the center position (black) and extreme field-of-view positions (red).

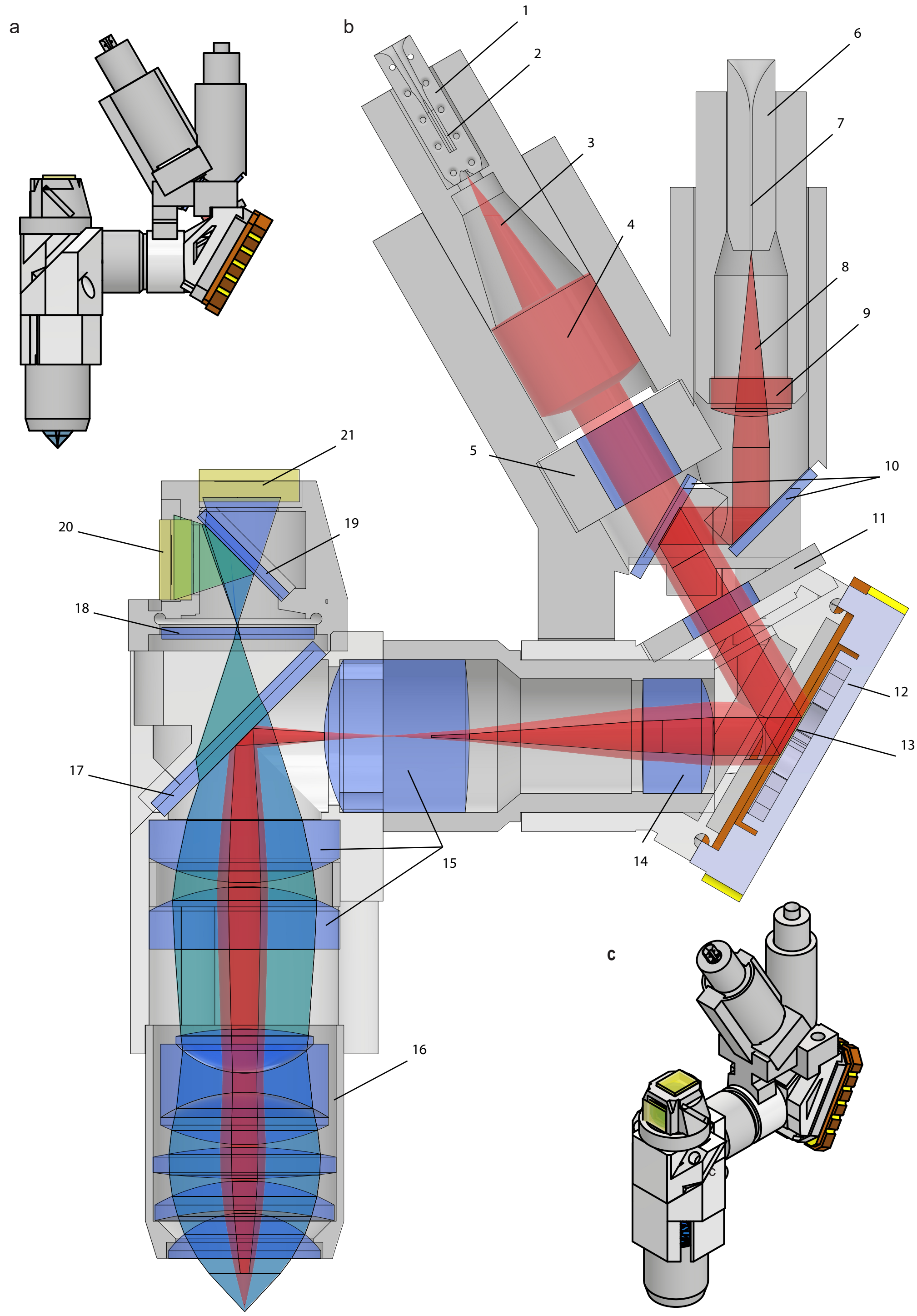

Supplementary Figure 2. Optical and Electronic parts for assembly of the miniature multiplane microscope. a, side-view rendered from CAD microscope assembly. b, section-view of the miniature multiplane microscope along the symmetry plane with the detail of the components: 1, 3D printed ferrule for two-photon excitation (2PE) fibers, 2, individual bore for a 2PE fiber defining the 3D position of the fiber tip, 3, laser cone leaving the fiber tip of a 2PE fiber with the NA predicted from its core size at 960 nm, 4, achromatic doublet collimation lens, 5, 4-element stack ETL (µT Lens) for adjusting the axial offset between the three-photon excitation (3PE) plane and the group of 2PE planes, 6, ferrule for 3PE fiber, 7, fiber bore of the ferrule for 3PE, 8, laser cone leaving the fiber tip of the 3PE fiber with the NA predicted from its core size at 1300 nm, 9, aspheric collimation lens, 10, dichroic mirrors combining 2PE path and 3PE path, 11, 1-element ETL for fine tuning for same field-of-view retrieval, 12, MEMS package, 13, MEMS scanner mirror, 14, scan lens from previous work[6], 15, compound tube lens composed of an achromatic lens and two plano-convex lenses, 16, objective lens from previous work[6], 17, dichroic mirror separating excitation optical path and emission optical path, 18, infra-red emission filter, 19, dichroic mirror separating blue light from (third-harmonic generation) and green fluorescence from GCaMP7f, 20, SiPM for green channel, 21, SiPM for blue channel. c, oblique-view rendered from CAD microscope assembly.

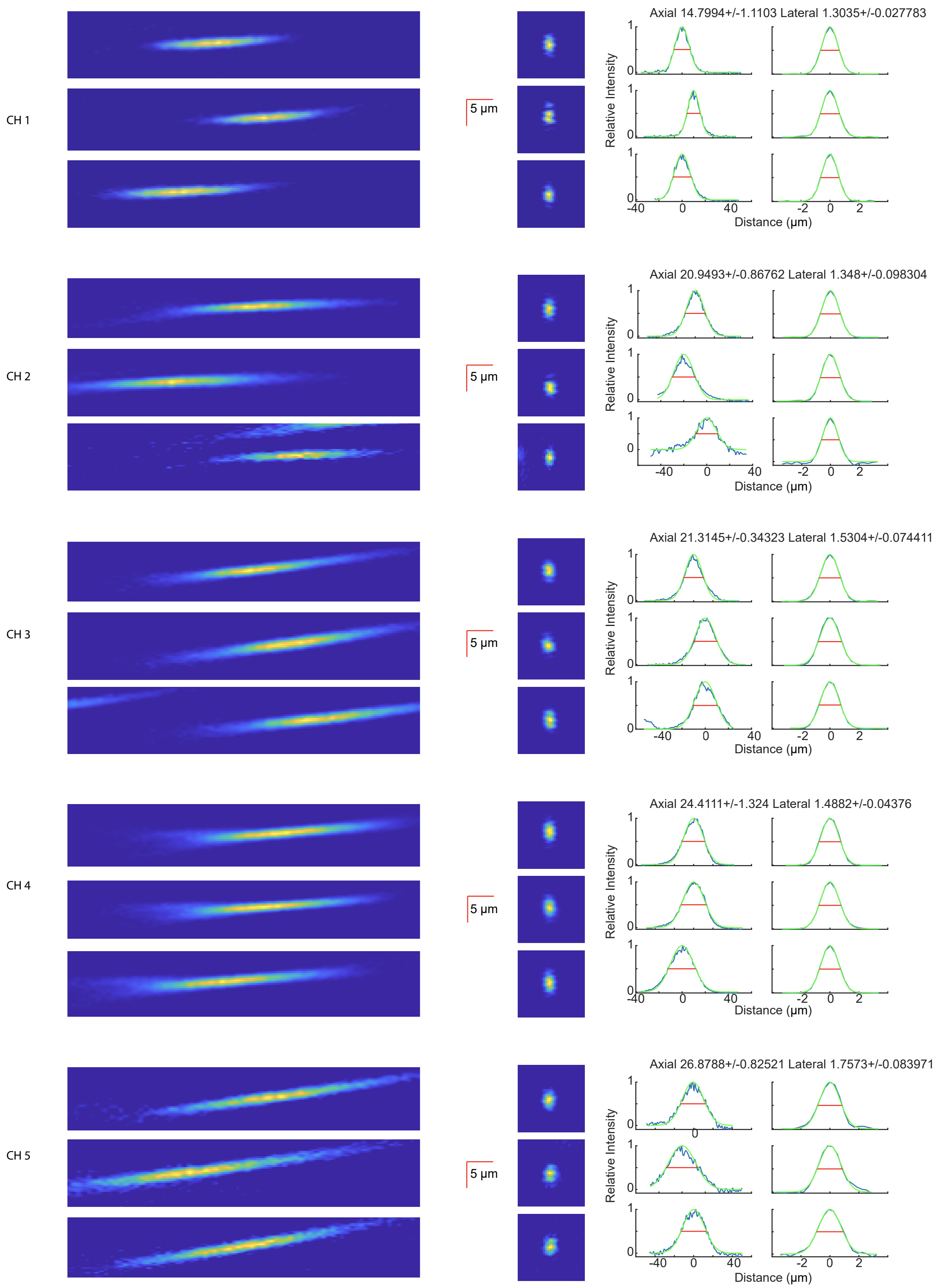

Supplementary Figure 3. Optical resolution characterization. Point spread function measurement projection on a lateral dimension (slow axis, left column), the axial dimension (second column from left), projections on the axial dimension (blue) with Gaussian fit (green, second column from right) and projection on the fast axis (blue) and its Gaussian fit (green, rightmost column). All five channels have been separately characterized with the channel 1 on the top row and up to the channel 5 in the bottom row.

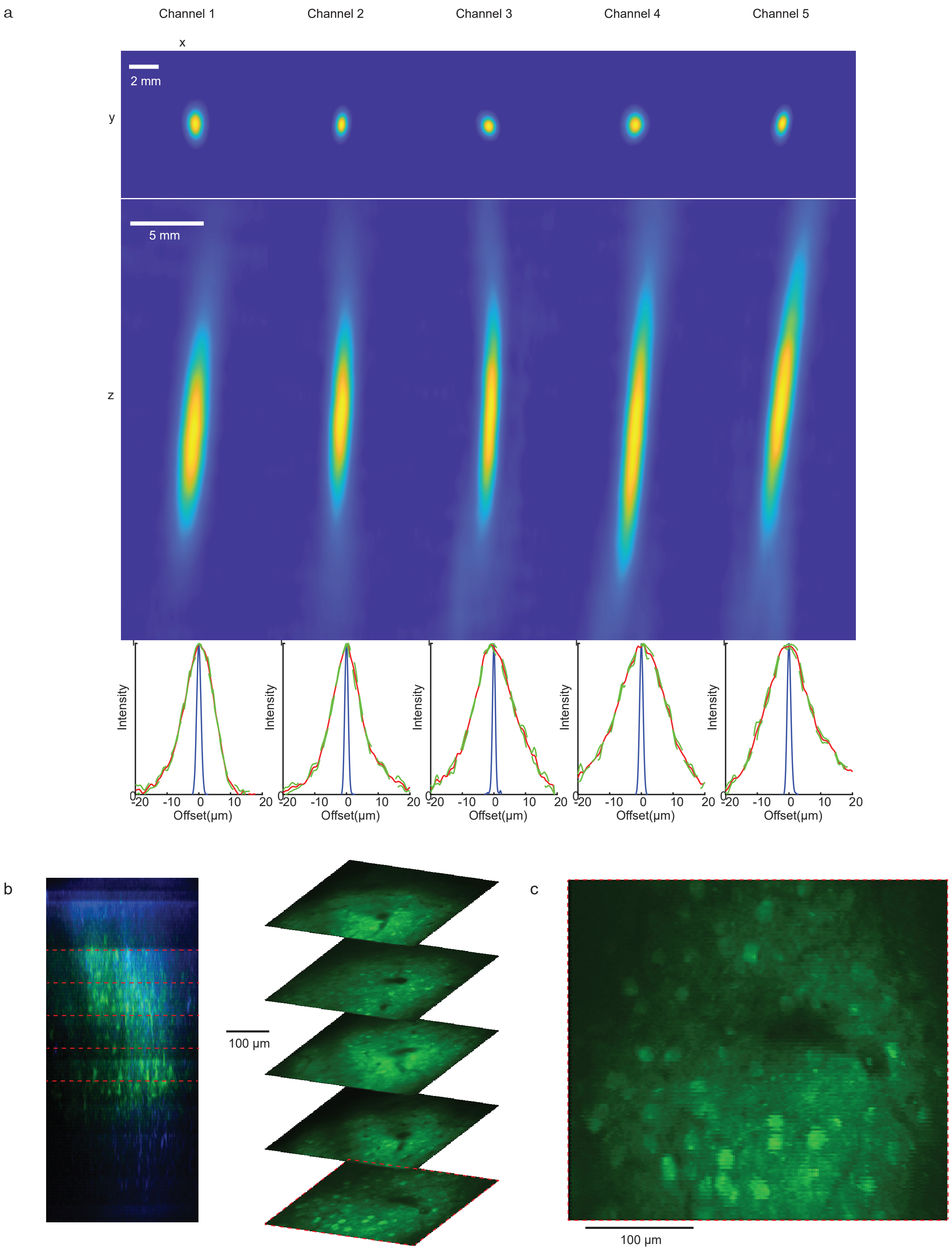

Supplementary Figure 4. High Speed Microscope version. a, Point spread function measurement projection on a lateral dimension (top row), the axial dimension (middle row) and corresponding profiles along a lateral dimension (blue) and axial dimension (red) (bottom row). All five channels have been separately characterized, from left to right, channel 1 to channel 5, respectively. b, Maximum lateral projection of a Z-stack collected using the three-photon channel and an external motor in steps of 5 μm (left column) with the depth of imaging planes labelled, with corresponding average images (right column). c, The deepest channel (three-photon excitation) average image, showing the image quality in the cortical layer 5.

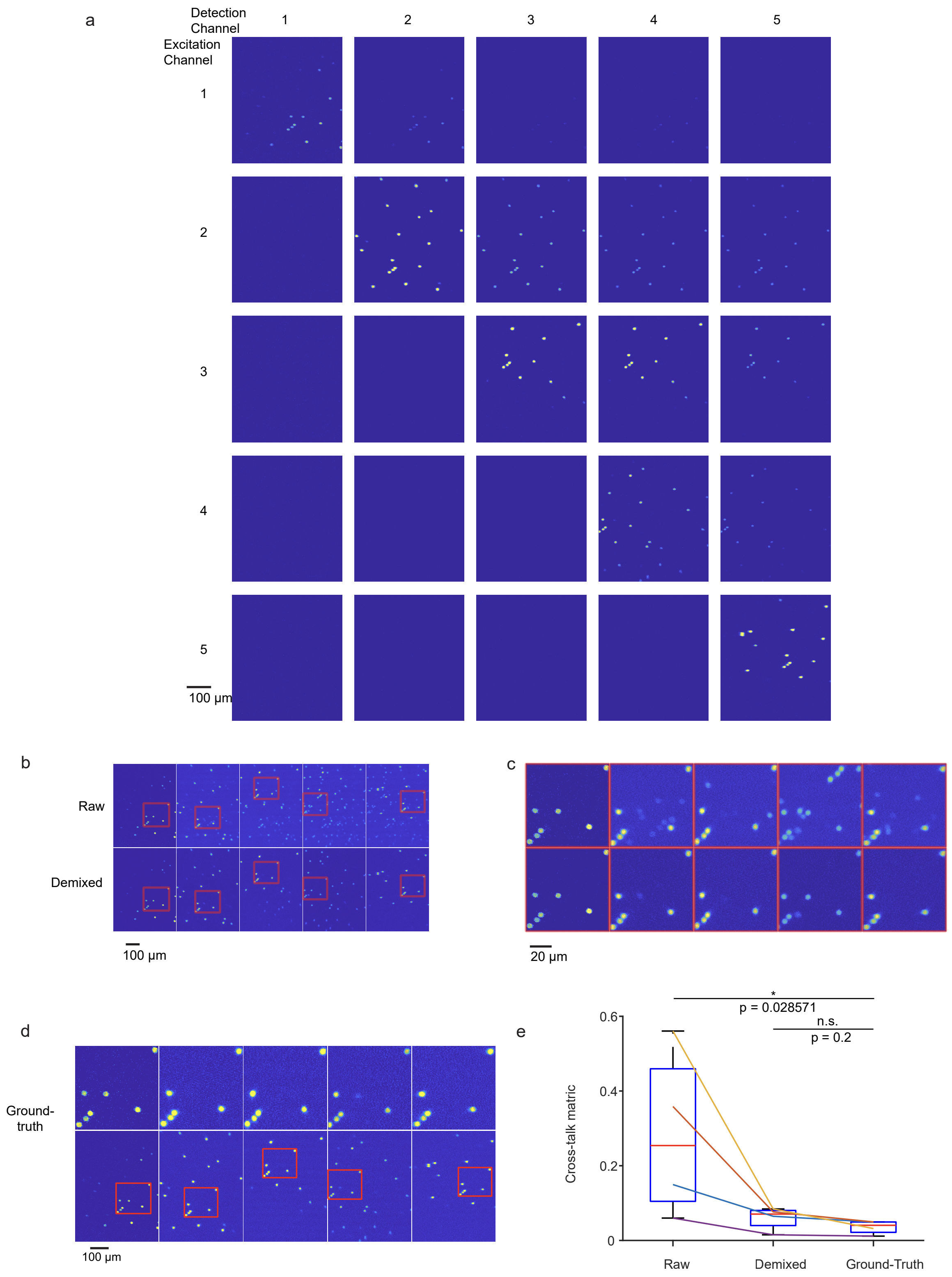

Supplementary Figure 5. Ground truth data for de-mixing algorithm. a, summary of the de-mixing ground-truth data with the axial projections of the imaged 10 µm bead volumes with the only exciting fiber operational on the rows and the recorded channels on the columns. Note that recorded channels are ordered in the electronic order, such that each next channel is ~ 8 ns later. On the diagonal we have the operational channel and the ground-truth signal and all the cross-talk is in the trailing channels, but never in the previous. b, axial projection of the same beads volume imaged as in a, but with all fibers operational. Raw data on the upper row and data after de-mixing algorithm in the bottom row. c, zoom on the same data as b, on the outlined in red areas, that were mapped in-between channel, where the same set of beads were found in each imaged volume. d, same axial projection from ground-truth data as in a, with the same beads outlined as in b and c and the corresponding zoom. e, cross-talk metric described in methods, that is the sum of absolute values of the signal in all detect ROIs for each channel, but computed in the trailing channels, to assess how much a given structure is producing signal in the trailing channels. Wilcoxon rank sum test was used to evaluate statistical significance with n = 4 channels per condition. Lines link individual points corresponding to channels 2-5, subject to cross-talk.

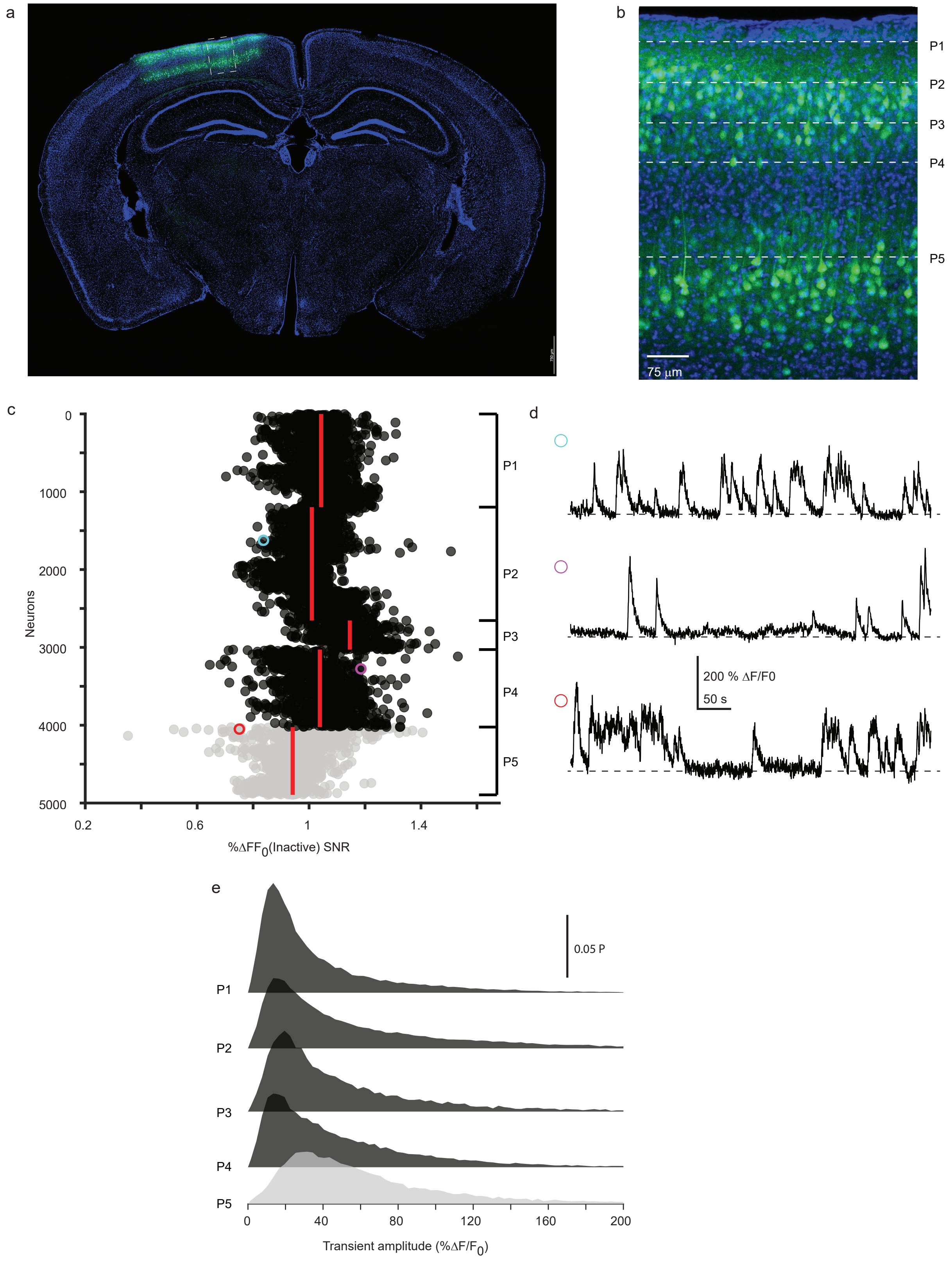

Supplementary Figure 6. Example cortical labelling from histology and comparison of neuronal signal to noise and transient amplitudes by imaging plane. a, example coronal section from one of the experiments in posterior parietal cortex showing DAPI staining of neuronal somata (blue) and neurons labelled with GCaMP7s (green). Dashed box outlines the region shown enlarged in b. b, enlarged region of the coronal section through posterior parietal cortex shown in a, with indication of the approximate locations of the five imaging planes (dashed lines, P1 to P5). P1 to P4 are the 2P excitation planes and P5 the 3P excitation plane. c, neuronal signal to noise ratio for all neurons recorded from all planes. Red lines denote the median signal to noise ratio for each imaging plane. Cyan, magenta and red outlined data points indicate the data points for the neurons for which example kinetic data are shown in d. Data from 4899 neurons, from 4 datasets from 4 mice. d, example kinetic data for the neurons indicated in c. e, distributions of Ca^{2+}-transient amplitudes for all neurons recorded in the five imaging planes (P1 to P5). Plane definitions and data as described in c.

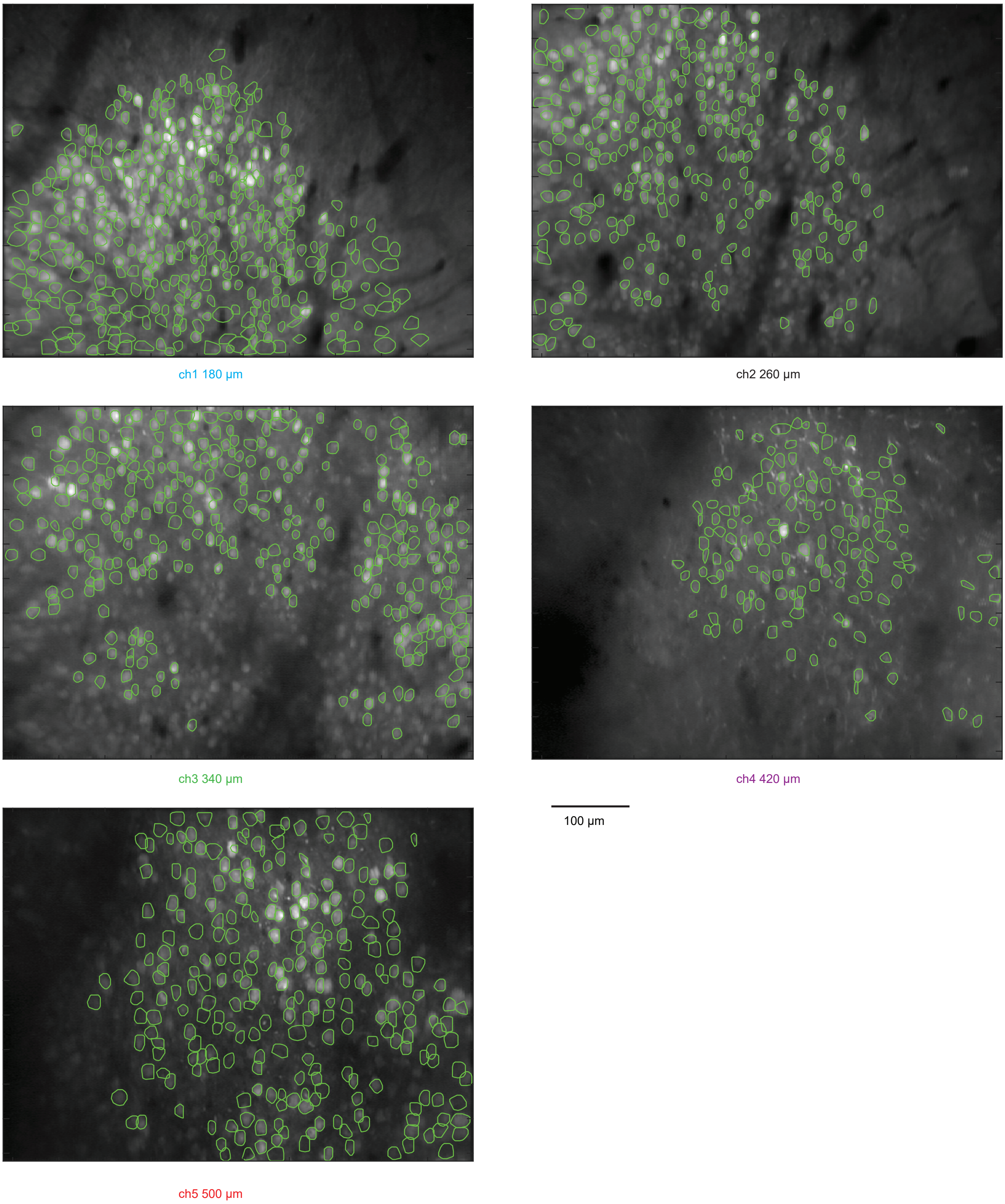

Supplementary Figure 7. Demonstration of the automated segmentation using Suite2p. Five average images corresponding to the field of view of each imaging channel, same data as in Fig. 2, showing regions of interest detected using Suite2p. The average images are displayed using a gamma transformation with gamma parameter of 0.5 for better visibility of dim neurons. The channels and imaging depth corresponding to each average image is annotated under each image.

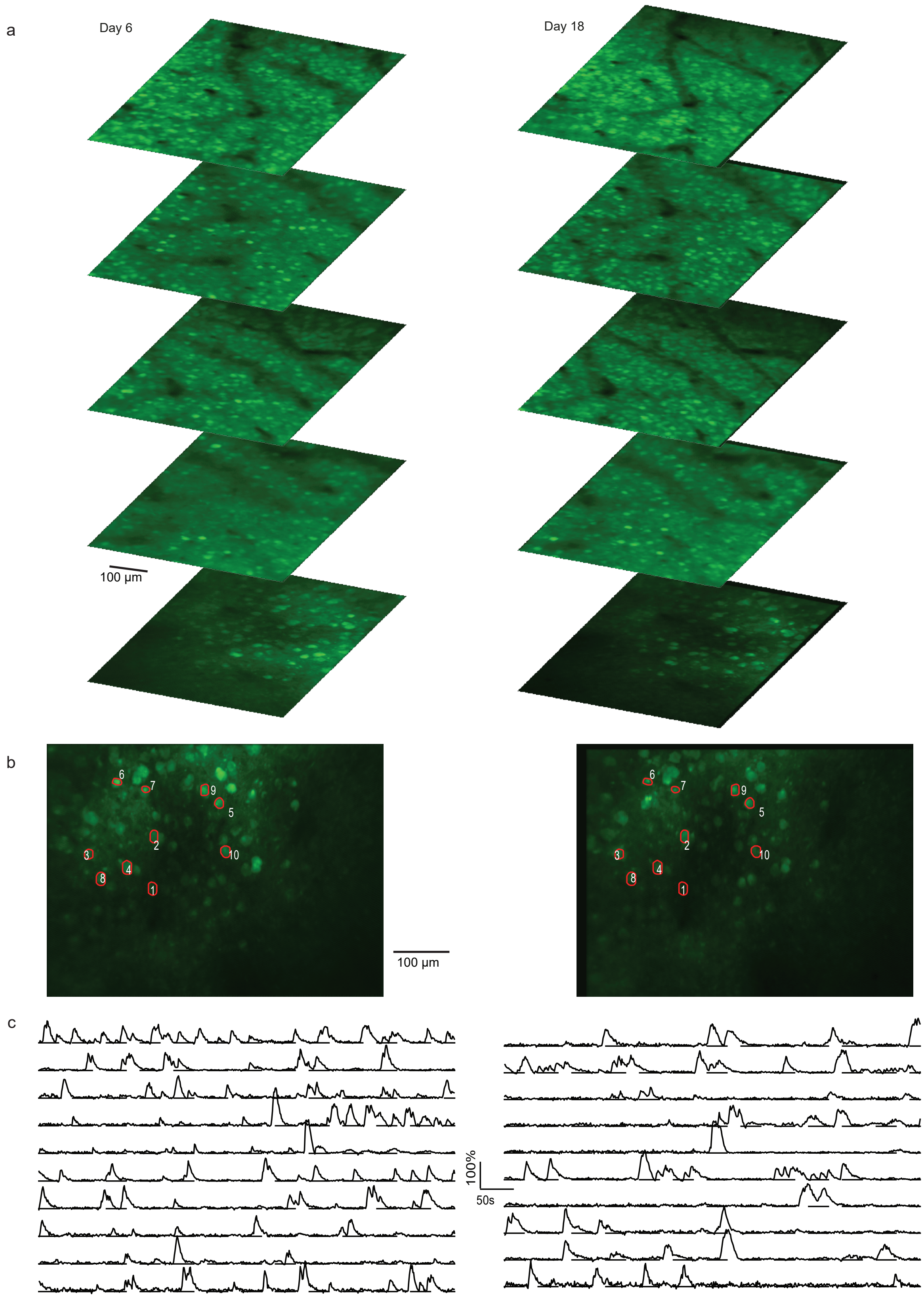

Supplementary Figure 8. Multiplane imaging is stable over weeks. a, overview average images from all planes of labelled neurons in the posterior parietal cortex on day six (left) and near identical populations imaged on day 18 (right). b, overview images from channel five (3PE) in a, showing 10 example ROIs from which fluorescence traces are shown in c. c fluorescence traces corresponding to the ROIs labelled in b.

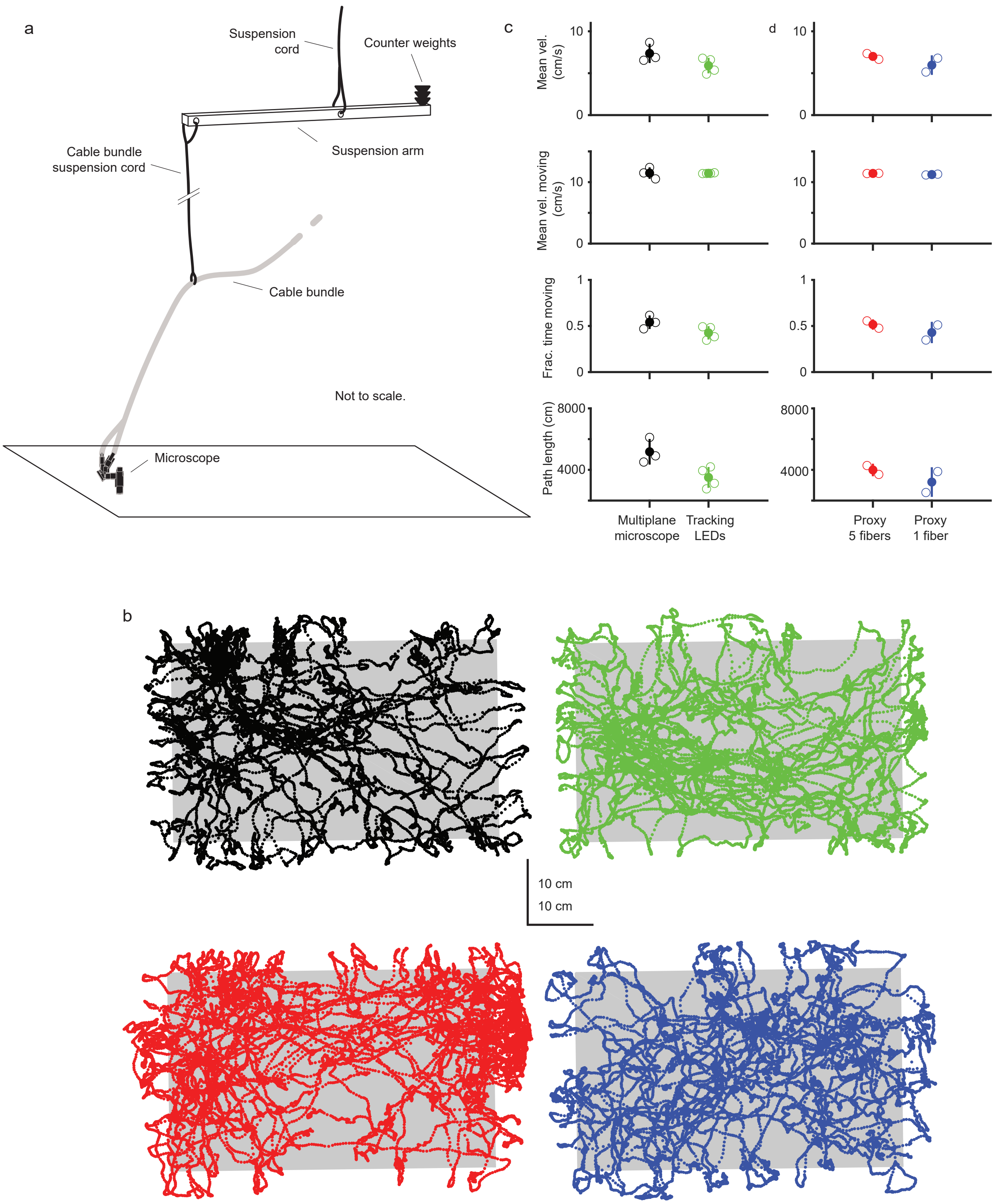

Supplementary Figure 9. Schematic illustration of counter-weight system and influence of microscope and cable bundle on behavioral parameters. a, schematic illustration of the counter-weight system used to reduce the influence of microscope and cable bundle on the animals behavioral performance. Note that the diagram is not to scale. b, example mouse trajectories for individual animals carrying the multiplane microscope (black, upper left), tracking LEDs only (green, upper right), weight-matched proxy microscope with a cable bundle including five optical fibers (red, lower left) and proxy microscope with a cable bundle including a single optical fiber (blue, lower right). Data in each panel from a 568 s dataset from one mouse. Scale bar applies to all panels. c, scatter plots of mean velocity (top panel), mean velocity when moving (second panel from top), fraction of time spent moving (third panel from top) and total path length when moving (bottom panel) for mice carrying either the multiplane microscope (black) or tracking LEDs only (green). Plot shows data from individual datasets (open circles) and mean and standard deviation (closed circle and vertical line). Each dataset was 568 s in duration, with data from 3 datasets from 3 mice for the multiplane microscope and 4 datasets from 3 animals for the tracking LEDs only. d, same as in c, but for animals carrying a proxy microscope with a cable bundle including five optical fibers (red) and a proxy microscope with a cable bundle including a single optical fiber (blue). Datasets were 568 s in duration, with data from 2 datasets from 2 animals in both cases.

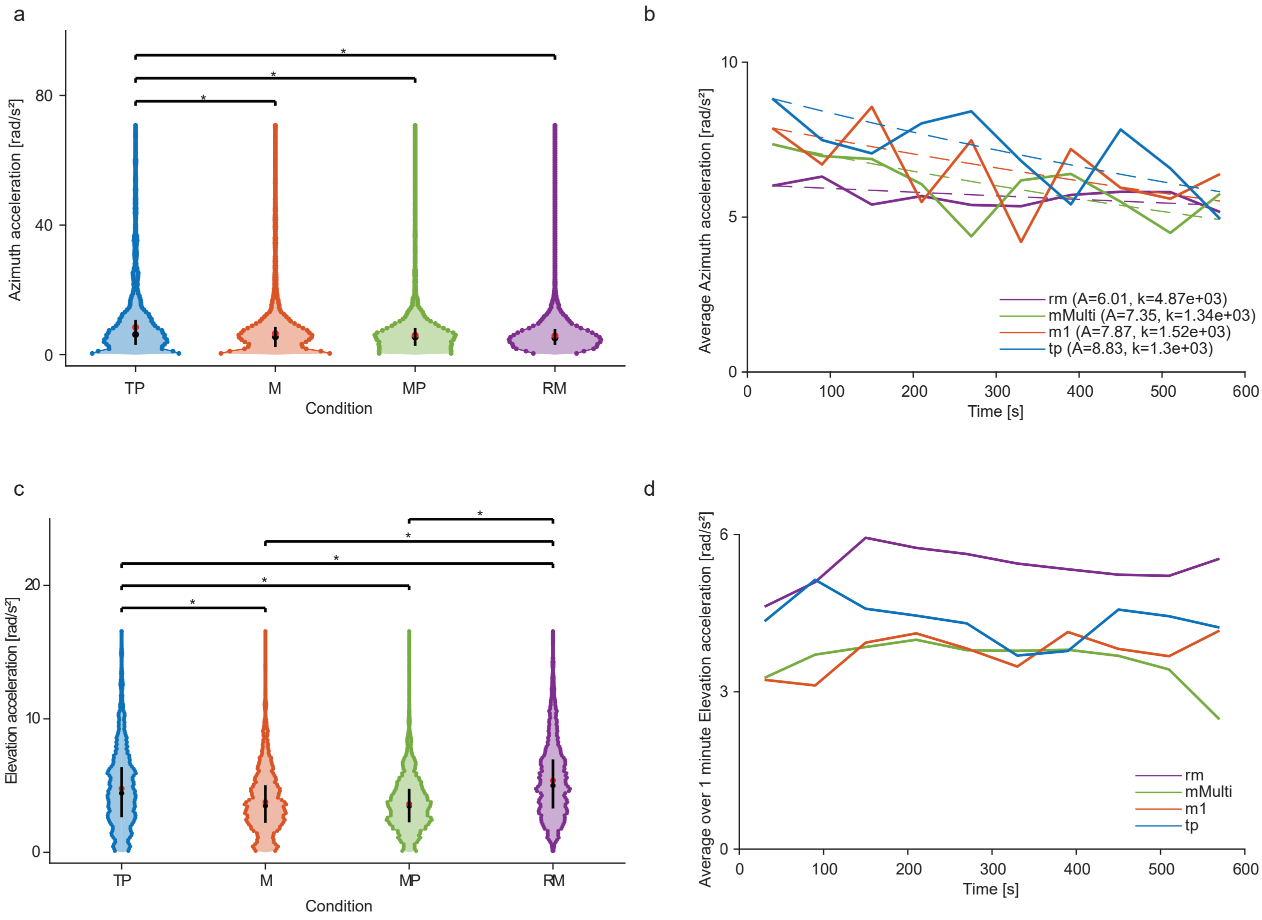

Supplementary Figure 10. Head elevation and azimuth accelerations comparison. a, distribution of average over 1 second absolute azimuth acceleration over 10 minutes. The four groups are TP (blue, tripod only, 4 datasets), M (orange, standard miniature microscope dummy with cabling, 2 datasets), MP (green, multiplane-like miniature microscope dummy with cabling, 2 datasets), RM (violet, real multiplane microscope, 3 datasets). The black bars denote 25th and 75th percentile, red circle is the mean, black circle is median). The side distributions of the violin plot are histograms of the distribution, probability density normalization. Full statistics and pairwise Wilcoxon's rank sum tests p-values are reported in Suppl. Tbl. 2. b, Average absolute azimuth acceleration over 10 minutes session (each data point is an average over 1 minute, over all datasets for each condition, same as in a). Coefficients of a single exponential decay are shown as indication of the trend, A – initial amplitude (equal to the first point) and k – inverse of the exponential constant in seconds. The fits are shown in dashed lines. Same color code as in a. c, d, Same as a, b with elevation instead of azimuth. Full statistics and pairwise Wilcoxon's rank sum tests p-values are reported in Suppl. Tbl. 3.

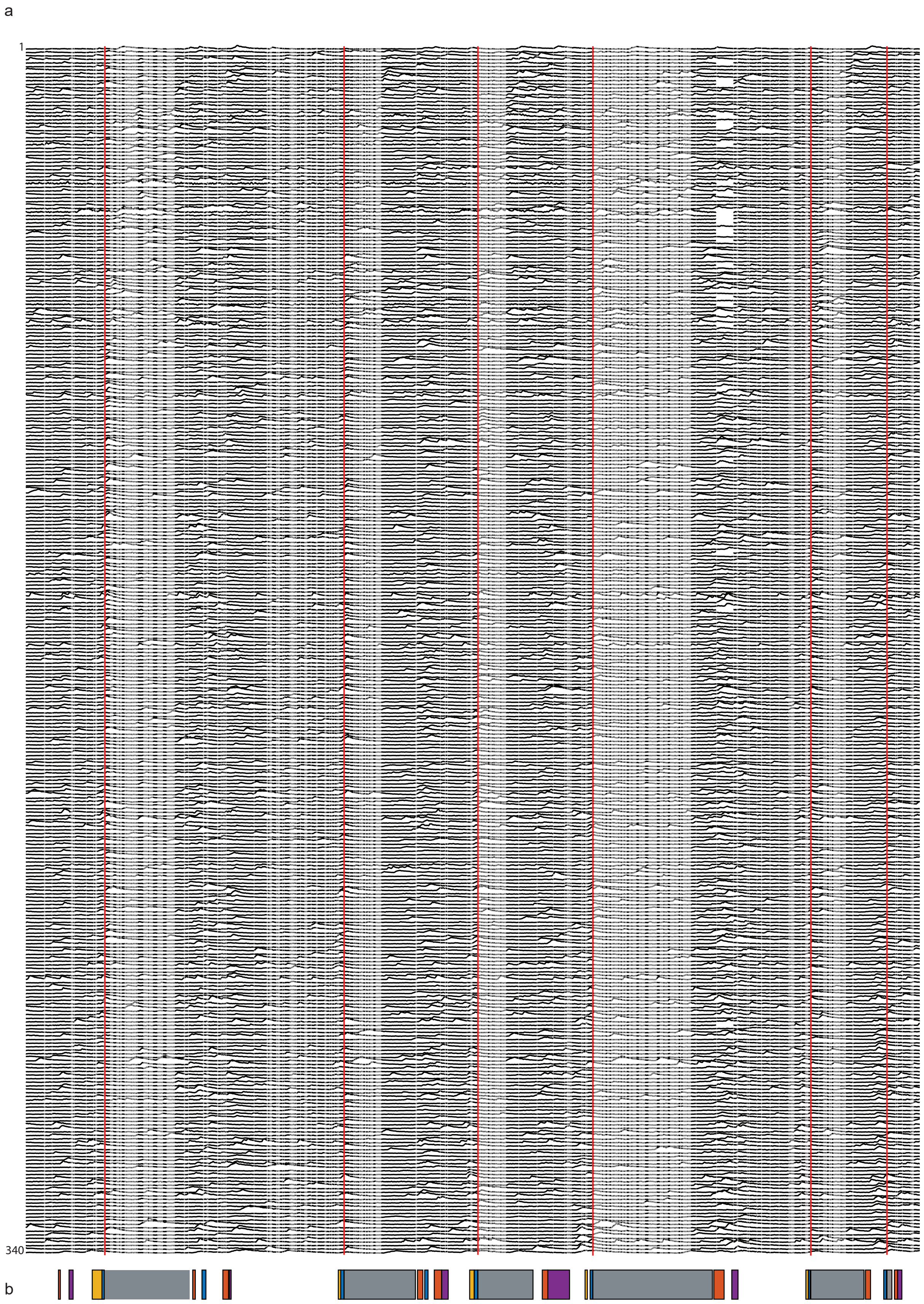

Supplementary Figure 11. Activity of all sequence neurons from an imaging session. a, fluorescence traces of all sequence neurons detected in the imaging session described in Fig. 4 (N = 340 neurons). Red lines label gap-crosses. b, temporal representation of identified behavioral events in the recording session aligned on the same x-axis as a, (blue, cross to top platform, orange, cross to middle platform, magenta, cross from middle platform to arena floor, gray, reward location on top platform).

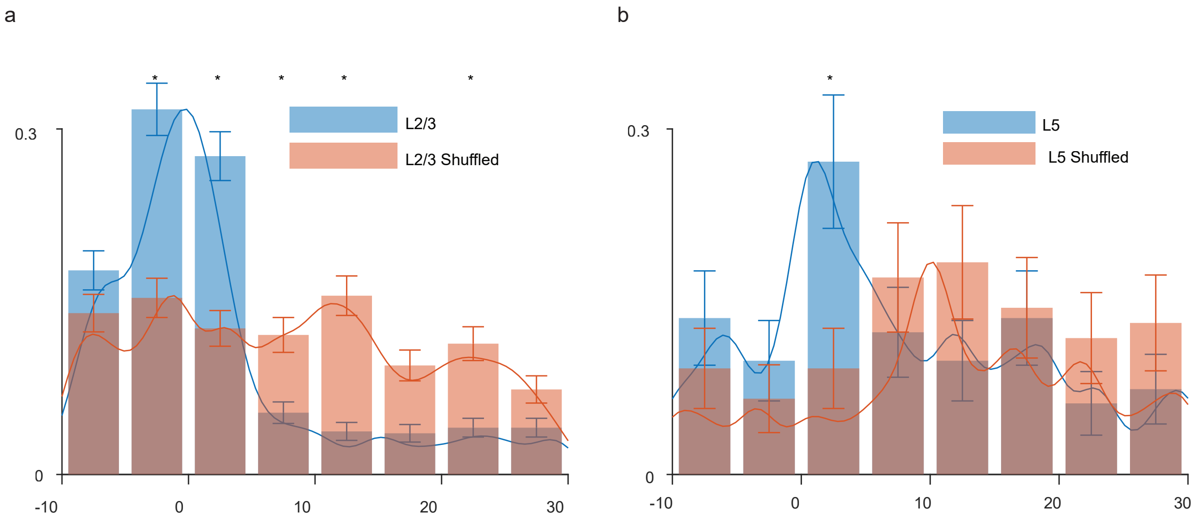

Supplementary Figure 12. Statistical comparison between temporal density of sequence cells in L2/3 and L5 with shuffled data. a, probability density histogram of sequence cells in L2/3 and L2/3 Shuffled data, where exactly the same method was applied to detect sequence cells, on the same data with the difference that Shuffled data had the behavior event times randomly chosen trough the imaging sequence. Error bars show ±SD assuming Poisson distribution of sequence cells occurrences within each time-bin. Spline distribution fits shown as alternative continuous representation (ksdensity Matlab function was used to produce the fit on the histogram counts data). Asterisks mark statistical significance of the difference of distribution assessed in each time-bin (see Methods and Suppl. Tbl. 4, for detail of statistical method). b, as in a but for sequence cells in L5 and L5 Shuffled data.

	Ref 7	Ref 8	Current study
**Lateral/Axial Resolution**	1.15±0.15 μm/17.80±0.85 μm or 1.21±0.13 μm/14.53±2.12 μm	0.68 μm/3.73 μm or 0.79 μm/7.04 μm or 1.46 μm/23.68 μm	1.45 ± 0.11 μm/22.2 ± 1.8 μm or 1.32 ± 0.20 μm/15.24 ± 3.31 μm
**Collection NA**	0.5	0.74 or 0.67 or 0.4	0.9
**Field of View**	0.5 × 0.5 mm² or 0.42 × 0.42 mm²	0.3 × 0.26 mm² or 0.5 × 0.425 mm² or 1 × 0.8 mm²	0.6 × 0.45 mm² or 0.3 × 0.26 mm²
**Pixel Resolution**	256 x 256	256 x 256	410 x 340 or 200 x 160
**Frame Rate**	15 Hz or 40 Hz	20 Hz	9.73 Hz or 40 Hz
**Axial Shift Range**	240 μm	None	up to 575 μm
**Multiple plane imaging**	15 Hz or 40 Hz divided by planes number	None	9.73 Hz or 40 Hz for all 5 planes
**Weight**	2.4g	2.6g	2.5g

Supplementary Table 1. Recent miniature microscopes. A table comparing characteristics of the multiplane microscope described here as well as the microscopes described in Ref 7 and Ref 8.

	Group	N	Mean	Median	Std	MAD	TP	M	MP	RM
1	TP	1117	8.4433	6.2422	8.9458	5.9161	NaN	4.2649e-05	2.6371e-05	4.4082e-05
2	M	1059	6.5364	5.5005	6.5915	4.1780	4.2649e-05	NaN	1	1
3	MP	1127	5.9910	5.4378	4.3970	3.2536	2.6371e-05	1	NaN	1
4	RM	1125	5.8515	5.1553	3.6947	2.8915	4.4082e-05	1	1	NaN

Supplementary Table 2. Statistics of azimuth angular acceleration behavior assay. Statistics for distributions in Suppl. Fig. 10a, including pairwise p-values of Kruskal-Wallis test.

	Group	N	Mean	Median	Std	MAD	TP	M	MP	RM
1	TP	1117	4.7731	4.4327	2.8630	2.2695	NaN	1.5087e-15	9.7810e-21	3.4446e-07
2	M	1059	3.7659	3.4881	2.2130	1.7400	1.5087e-15	NaN	1	1.0339e-44
3	MP	1127	3.6213	3.4616	1.9192	1.5177	9.7810e-21	1	NaN	5.4916e-56
4	RM	1125	5.3819	5.0029	2.7330	2.1763	3.4446e-07	1.0339e-44	5.4916e-56	NaN

Supplementary Table 3. Statistics of elevation angular acceleration behavior assay. Statistics for distributions in Suppl. Fig. 10c, including pairwise p-values of Kruskal-Wallis test.

	L2/3	L2/3 Shuffled	L23 vs Shuffled p-value	L5	L5 Shuffled	L5 vs Shuffled p-value
-10 : -5s	109±10.44	74±8.6	0.23448	11±3.32	7±2.65	0.38333
-5 : 0s	195±13.96	81±9	0.0091804	8±2.83	5±2.24	0.41212
0 : 5s	170±13.04	67±8.19	1.5585e-08	22±4.69	7±2.65	0.018176
5 : 10s	33±5.74	64±8	0.047093	10±3.16	13±3.61	0.14502
10 : 15s	23±4.8	82±9.06	9.0271e-05	8±2.83	14±3.74	0.52612
15 : 20s	22±4.69	50±7.07	0.63391	11±3.32	11±3.32	0.16181
20 : 25s	25±5	60±7.75	0.0078034	5±2.24	9±3	0.68283
25 : 30s	25±5	39±6.24	0.1407	6±2.45	10±3.16	0.64286

Supplementary Table 4. Summary of sequence neurons counts evolution through the behavioral epoch around the gap crossing in L2/3 and L5. Summary of statistical data for histogram shown in Suppl. Fig. 12a,b, for L2/3 and L5 with their shuffled versions (format M±SD of the counts, each count being a detected sequence neuron, for probability density see Suppl. Fig. 12a,b). To assess statistical significance, the difference between neighbouring onset-times distribution was considered (inverse of distribution measures of instantaneous density). Wilcoxon rank sum test was used to assess significance of difference between medians of each bin of L2/3 and L5 data against the shuffled versions and each p-value is reported in a separate column. A threshold of 0.05 was set for statistical significance and marked by an asterisk in Suppl. Fig. 12a,b.

Supplementary References

1 Klioutchnikov, A. *et al.* A three-photon head-mounted microscope for imaging all layers of visual cortex in freely moving mice. *Nat Methods* **20**, 610–616 (2023). https://doi.org/10.1038/s41592-022-01688-9

2 Zong, W. *et al.* Large-scale two-photon calcium imaging in freely moving mice. *Cell* **185**, 1240–1256 e1230 (2022). https://doi.org/10.1016/j.cell.2022.02.017

3 Wu, R. *et al.* A versatile miniature two-photon microscope enabling multicolor deep-brain imaging. *Nat Methods* **22**, 1935–1943 (2025). https://doi.org/10.1038/s41592-025-02780-6
